# The interplay between metabolic disorders and tendinopathies: Systematic review and meta‐analysis

**DOI:** 10.1002/jeo2.70429

**Published:** 2025-09-10

**Authors:** Paola De Luca, Giulio Grieco, Silvia Bargeri, Cecilia Colombo, Stefania Guida, Michela M. Taiana, Laura de Girolamo

**Affiliations:** ^1^ IRCCS Ospedale Galeazzi Sant'Ambrogio, Orthopaedic Biotechnology Laboratory Milan Italy; ^2^ IRCCS Ospedale Galeazzi Sant'Ambrogio, Unit of Clinical Epidemiology Milan Italy

**Keywords:** diabetes, dyslipidaemia, metabolic alteration, tendinopathy

## Abstract

**Purpose:**

To evaluate the prevalence of tendinopathies in relation to metabolic factors and to investigate associations between tendinopathies and metabolic conditions.

**Methods:**

The current review synthesised screened articles in accordance with the preferred reporting items for systematic reviews and meta‐analyses guidelines predesigned criterion which were from PubMed, Scopus and Web of Science published up to 31 March 2024. Eligible studies included cohort, case‐control or cross‐sectional designs.

**Results:**

Fifty‐three studies were included. Achilles tendinopathy emerged as highly prevalent in diabetic individuals (6%; 95% confidence interval [CI]: 42%–91%). Moreover, diabetes was identified as a risk factor for Achilles tendinopathy development with an odds ratio (OR) of 7.22 (95% CI: 2.61–19.97). Diabetes was also linked to upper limb tendinopathies, including medial epicondylitis (OR: 11.27, 95% CI: 2.01–63.02) and trigger finger (OR: 3.79, 95% CI: 1.87–7.65). A pooled prevalence estimate found that 13% (95% CI: 4%–21%) of tendinopathy patients had hypercholesterolaemia and a prevalence of 38% (95% CI: 5%–80%) of tendinopathy was found among statin users. However, high study heterogeneity limited the reliability of these two findings. Even if body mass index alterations were observed in tendinopathy patients with a pooled prevalence of 64% (95% CI: 62%–66%), a causation could not be definitively established. The analysis of the impact of sex exhibited men higher rates of tendon pathology associated with dyslipidaemia, whereas diabetic women demonstrated a greater prevalence of trigger finger tendinopathy.

**Conclusion:**

Results showed that diabetes, dyslipidaemia and obesity contribute to the tendinopathies development, highlighting their multifactorial aetiology with sex differences influencing specific pathologies. These findings suggested periodic metabolic evaluations in susceptible individuals to tendon overload, chronic pain or recurrent injuries.

**Level of Evidence:**

Level III.

## Graphical abstract

This systematic review highlights a strong link between metabolic conditions and tendinopathies. Diabetes significantly increases the risk, especially for Achilles and upper limb tendinopathies. Dyslipidemia, statin use and body mass index changes also show associations, though evidence varies. Sex differences were observed in disease patterns. These findings support incorporating metabolic evaluations into the clinical assessment of individuals at risk for tendon overload or recurrent injuries.

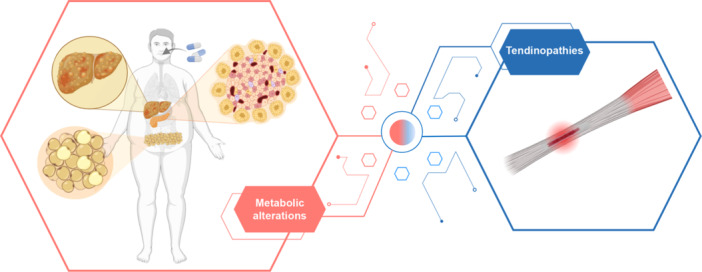

**Abbreviations**
AGEs: advanced glycation end‐products
BMI: body mass index
CI: confidence interval
MeSH: medical subject headings
MetS: metabolic syndrome
MOOSE: meta‐analyses of observational studies in epidemiology
MRI: magnetic resonance imaging
NOS: Newcastle‐Ottawa scale
OR: odds ratio
PRISMA: preferred reporting items for systematic reviews and meta‐analyses
QUIPS: quality in prognosis studies
RR: risk ratio
TSPCs: tenocytes and tendon stem/progenitor cells

## INTRODUCTION

Tendinopathy is a highly prevalent musculoskeletal pathology [7] with a significant socioeconomic impact [7, 21]. The aetiology of tendinopathy is multifactorial and remains poorly understood, even if there is a growing evidence that suggests the contribution of systemic and metabolic factors, including environment, genetics, demographics and pharmacological interventions, in influencing the onset, progression and severity of tendinopathy.

Metabolic dysfunctions have increasingly been implicated in tendinous alterations by causing systemic inflammation, microvascular complications and alterations to tendon homeostasis.

Recent studies have shown a clear link between obesity and the onset and progression of tendinopathy [42] both for the increased mechanical loading on tendons and for obesity‐induced systemic inflammation, which is mediated by elevated pro‐inflammatory cytokines. Previously was demonstrated that diabetes is linked to a threefold higher risk of tendinopathy, while tendinopathy increases diabetes risk by 1.3 times [57]. Diabetes also disrupts tendon healing by impairing tenocyte and tendon stem cell function through hyperglycaemia and advanced glycation end products, which reduce cell viability and function [71].

Similarly, insulin resistance and lipid metabolism disturbances such as hypercholesterolaemia, have been shown to compromise the structure of tendon collagen and repair capacity, further increasing susceptibility to tendinopathy [68, 69].

The link between lipid‐lowering drugs and tendinopathy is still being debated. Some studies suggest that statins may increase the risk of tendinopathy [30, 68], a theory supported by laboratory data indicating increased matrix metalloproteinase activity and tendon matrix breakdown [53]. However, others report a protective effect of statins [40]. Despite emerging evidence linking metabolic dysfunctions to tendon pathology, the current literature presents inconsistent findings, with significant heterogeneity in study designs, populations and diagnostic criteria.

Data also show sex differences in tendon structure, blood flow and healing, which are shaped by hormones, genes and mechanics and which may affect risk [60]. Therefore, further investigation into the influence of sex in tendinopathies becomes opportune.

This systematic review and meta‐analysis aims to synthesise available clinical evidence on the relationship between several metabolic alterations and tendinopathy in different anatomical sites. By providing a comprehensive analysis of the metabolic and sex‐related factors underlying tendinopathy to determine whether a statistically significant relationship exists, this work aims to inform future research directions and clinical strategies for managing this disabling condition in consideration of the different patient characteristics.

## MATERIALS AND METHODS

This systematic review was reported in accordance with the preferred reporting items for systematic reviews and meta‐analyses (PRISMA) and meta‐analyses of observational studies in epidemiology (MOOSE) guidelines [50, 56, 65]. The study protocol was registered with the International Prospective Register of Systematic Reviews (PROSPERO) on 11 March 2024 (CRD42024523183).

The primary aims were:

1) to assess the prevalence of the most common metabolic alterations among patients with any type of tendinopathy, and vice versa.
2) to assess the association (in terms of odds ratios) between metabolic alterations and tendinopathy in studies comparing patients with and without metabolic alterations or with and without tendinopathies.

The secondary aim was to analyse the impact of sex (i.e., being a male or a female) on metabolic parameters as modifiers of the risk to develop tendinopathy.

### Eligibility criteria

We included primary studies that examined either the relationship between: (i) tendinopathy in patients with or without metabolic alterations, or (ii) metabolic alterations in patients with or without tendinopathy.

Any type of tendinopathy was considered, excluding traumatic tendon ruptures. Tendinopathy diagnosis had to be based on factors such as pain severity, impaired load‐bearing capacity or imaging [48].

Metabolic alterations considered were: diabetes (type 1 and type 2), dyslipidaemia (hypercholesterolaemia and hypertriglyceridemia), body mass index (BMI) alteration (overweight and obesity) and metabolic syndrome (MetS), which had to be diagnosed through blood tests (e.g., blood sugar, cholesterol and triglycerides) [73] or anthropometric measurements (e.g., height, weight and body circumference) [37]. Studies involving patients using statins were also included, as statin use has been linked to the development of tendinopathy [39].

Any primary studies (e.g., case‐control, cross‐sectional and cohort studies) that investigated the relationship between tendinopathy and metabolic alterations, with or without a control group was eligible. Specifically, studies had to report either prevalence (e.g., prevalence of tendinopathy in metabolic patients) or association measures such as odds ratio (OR), risk ratio (RR) or hazard ratio (HR).

Case series, case reports, narrative reviews, notes and book chapters, studies involving animal models or in vitro experiments were excluded. No restrictions on publication year were applied.

### Search strategy

Scopus, PubMed and Web of Science electronic databases were interrogated for eligible studies published to 31 March 2024. The specific search strategies were created by an expert researcher in systematic review considering both MeSH terms and free text related to metabolic diseases and tendinopathy. The search strategy was adapted to the syntax of other databases and is presented in Appendix S1. In addition to the database search, reference lists of included studies and relevant reviews were manually reviewed to identify additional studies for inclusion.

### Study selection

Two couples of independent reviewers (P.D., M.M.T., G.G. and C.C.) screened independently all records by title and abstract of studies, and full‐text articles were selected if the inclusion criteria are met. Disagreement was resolved by consultation with a third couple (S.B. and S.G.).

Rayyan software (https://www.rayyan.ai) was used to manage all records and removing duplicates.

### Data extraction and analysis

Four reviewers (P.D., M.M.T., G.G. and C.C.) extracted on an Excel sheet the following data: characteristics of participants (e.g., age, sex, type of metabolic alterations, tendon studied, method of diagnosis of tendinopathy; characteristics of study design (e.g., country of enrolment institution, funding) and outcome data (e.g., n/N). Specifically, for estimating prevalence, the number of patients with tendinopathy or metabolic alteration was extracted out of the total number of patients included (e.g., N of patients with metabolic alterations recruited). For estimating association's measures, the number of patients with a condition (i.e., number of patients with tendinopathy in patients with and without metabolic alterations) out of the total number of patients included in each cohort (i.e., number of patients with and without metabolic alterations recruited). If a study did not report outcome data, the Authors were asked by email for further details. In case of missing outcome data, results were reported as narrative synthesis.

Disagreements were resolved by discussion between the two couple of reviewers (SB, SG) or with the involvement of a third couple (P.D., M.M.T., G.G. and C.C.).

### Risk of bias assessment (ROB)

Two independent reviewers (G.G. and P.D.) assessed the risk of bias, and any discrepancies were resolved through consultation with a third author (M.M.T. or C.C.). In order to evaluate the quality of cross‐sectional, cohort and case‐control studies, the Newcastle‐Ottawa scale (NOS) tool was employed to define these study designs as good, fair, or poor quality [74]. The quality in prognosis studies (QUIPS) tool was employed through classification as low, moderate, or high risk for the purpose of assessing the quality of prospective studies that specifically assess association outcome data prospectively [25] (Tables S4 and S5).

### Data synthesis and statistical analysis

Descriptive statistics were used to summarise the general characteristics of the included studies.

To assess prevalence, we restricted the analysis to cross‐sectional studies, as these are specifically designed to evaluate prevalence by capturing a ‘snapshot’ of data at a single point in time. A proportional meta‐analysis was conducted to estimate the prevalence, with 95% confidence intervals (CIs), of various types of tendinopathy (e.g., upper limb and lower limb tendinopathies) in patients with metabolic alterations. Similarly, we estimated the prevalence of metabolic alterations in patients diagnosed with tendinopathy. For each subgroup, the pooled prevalence estimate using the ‘metaprop’ command in Stata software was calculated [74]. To assess associations, data from all observational studies that contained a healthy control group (e.g., studies comparing patients with and without metabolic alterations or patients with and without tendinopathy) were included. A random‐effects meta‐analysis was performed using the Mantel‐Haenszel method with the ‘metan’ command in Stata software to calculate pooled ORs [23]. The ORs quantified the association between tendinopathy and metabolic alterations, both for the likelihood of tendinopathy in patients with metabolic alterations and vice versa. Results were considered statistically significant when the 95% CI for ORs did not include 1.

Subgroup analyses were performed as described in Table S1.

Heterogeneity between studies was assessed with Higgins *I* ^2^ defined as low if *I* ^2^ < 25%, moderate if between 25% and 50%, and substantial if >50% [26, 74].

### Equity, diversity and inclusion statement

Our author group includes six women and one man, comprising three PhD students, one early to mid‐career researcher, and three senior researchers. Our research team, though based at the same institution, brings diverse expertise in medical research, epidemiology and population health. Our search approach was broad and inclusive, without restrictions on gender, nationality, cultural background, language or age.

## RESULTS

### Search yield

**Figure 1:**
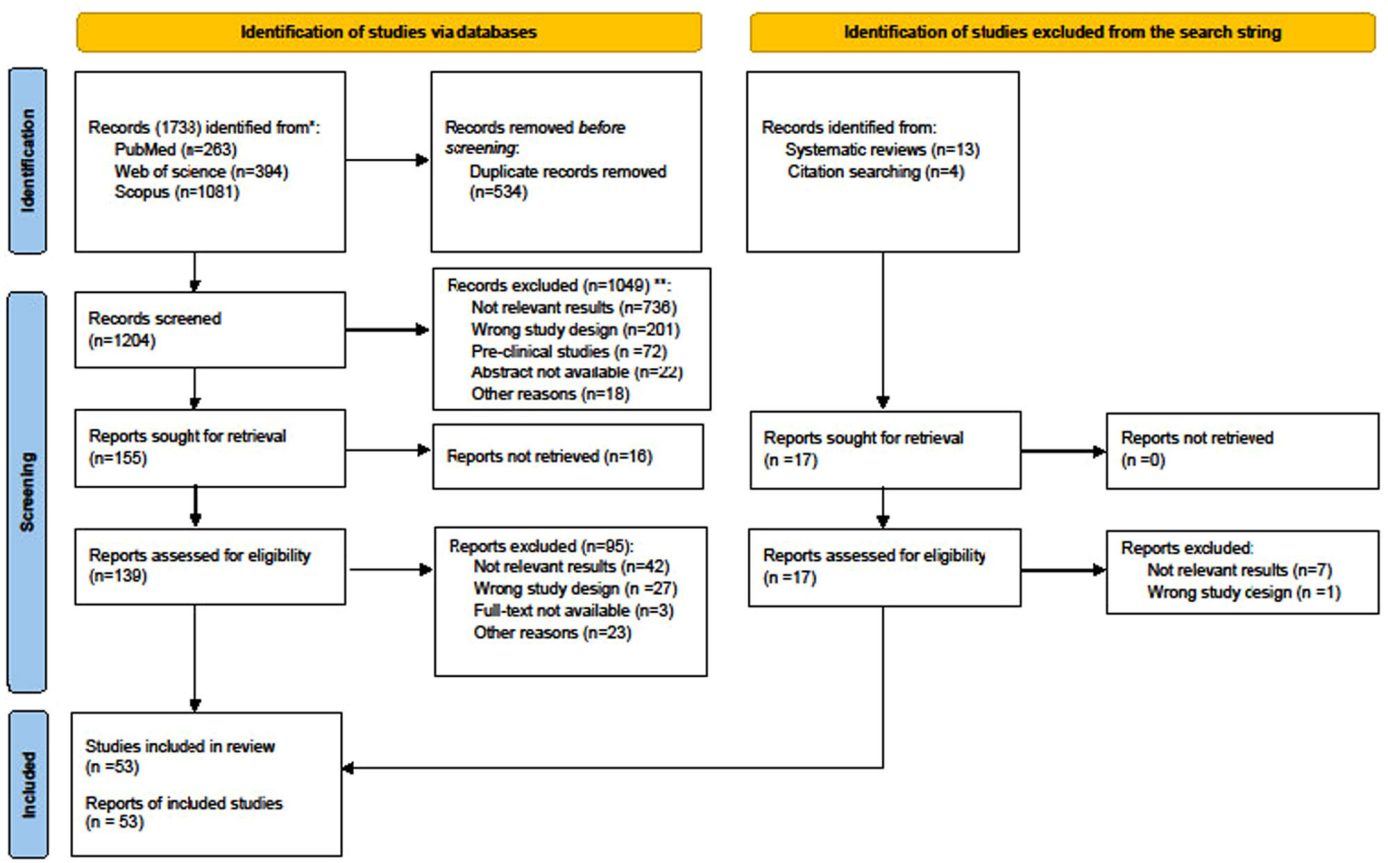
PRISMA 2020 flow diagram. PRISMA, preferred reporting items for systematic reviews and meta‐analyses.

The electronic search identified 1738 papers (Figure 1). After duplicates removal and screening by title and abstracts, the remaining 139 studies underwent a full‐text assessment, which resulted in the exclusion of 95 papers. Reference list checking of systematic reviews included in the search and citation searching led to identification of a further 17 papers suitable for inclusion of which only nine papers satisfied eligibility criteria. Finally, a total of 53 studies were included for review.

### Characteristics of included studies

Eighteen studies were case‐control studies, 24 were cross‐sectional studies and 11 were cohort studies. Among these studies, 28 recruited participants with metabolic disease (Table S2) and 25 patients with tendinopathy as primary outcome (Table S3). Within the first group of studies (28), 11 assessed tendinopathy by imaging evaluation (MRI, ultrasonography and echography), 14 by clinical examination, 2 by database/ICD‐10 screening and 1 not reported diagnosis method. In the second group of studies (25), 8 assessed tendinopathy by imaging evaluation, 13 by clinical examination, 1 by database screening and 3 not reported diagnosis method. The country of enrolment institution as well as the eventual funding was also reported (Tables S2 and S3).

### Quality assessment

All included trials were assessed using the NOS; for one cohort study assessing the outcome prospectively [25], the QUIPS tool was used to assess risk of bias, presenting an overall judgement of low risk of bias. In the metabolic disease group, the majority of the papers (*n* = 17; 61%) were rated as poor quality. In the tendinopathy group, fewer than half of the studies (*n* = 10; 40%) were rated as good quality. Further details are reported in Tables S4 and S5.

## META‐ANALYSIS

### Prevalence of tendinopathies in patients with metabolic alterations

**Figure 2:**
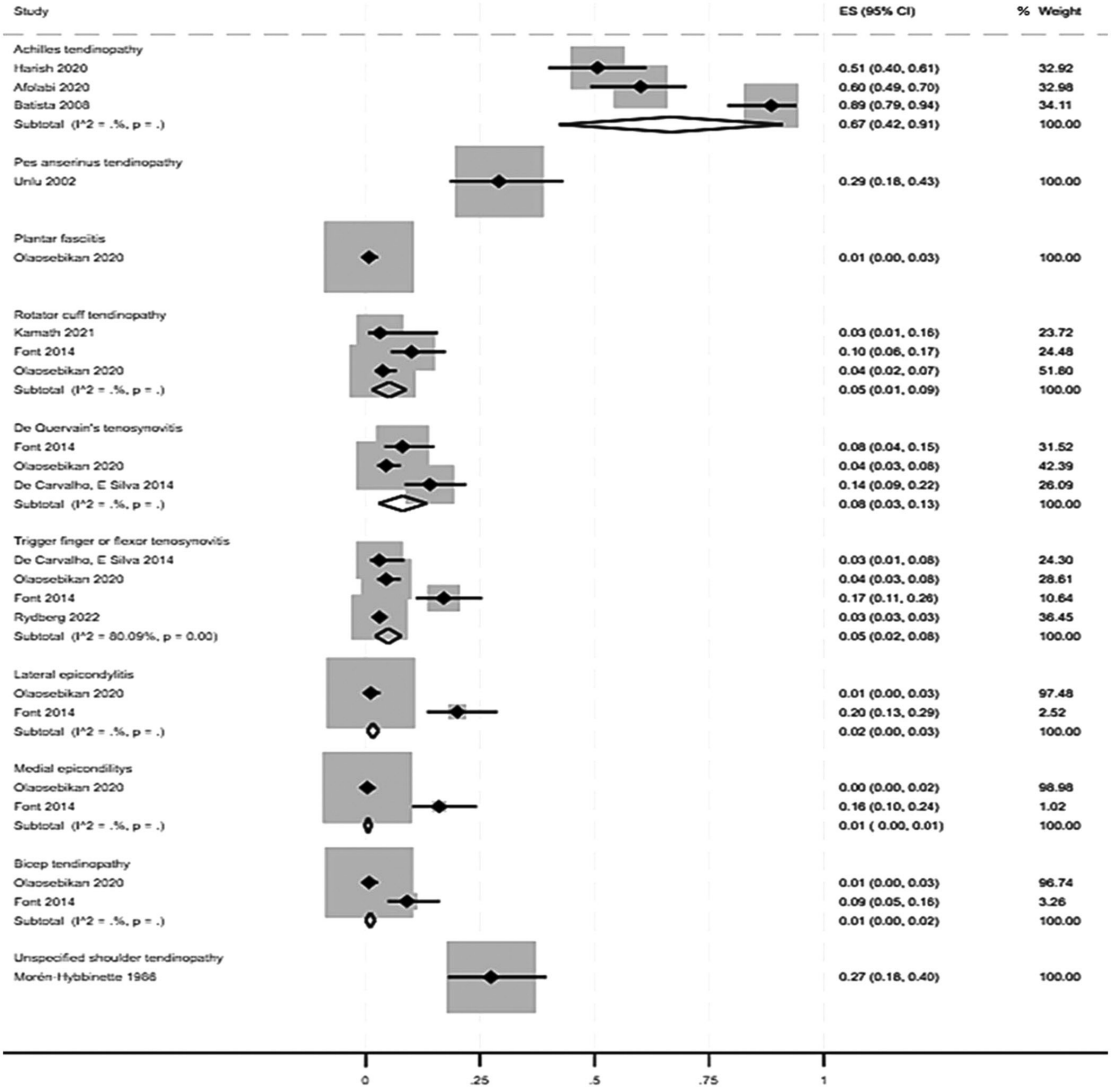
Prevalence of different tendinopathies in patients with diabetes derived from 10 cross‐sectional studies [4, 11, 14, 19, 22, 32, 51, 59, 71].

In patients with diabetes, the results showed an overall prevalence of lower limb tendinopathy ranging from 1% (95% CI: 0%–3%) [52] for plantar fasciitis to 67% (95% CI: 42%–91%) [4, 11, 22] for Achilles' tendinopathy. Likewise, the prevalence of upper limb tendinopathies ranged from 1% (95% CI: 0%–1%) [19, 52] for medial epicondylitis to 8% for De Quervain's tenosynovitis (95% CI: 3%–13%) [14, 19, 52]. One study [51] assessed rotator cuff tendinopathy without distinguishing subtype, reporting a prevalence of 27% (95% CI: 18%–40%) as reported in Figure 2.

**Figure 3:**
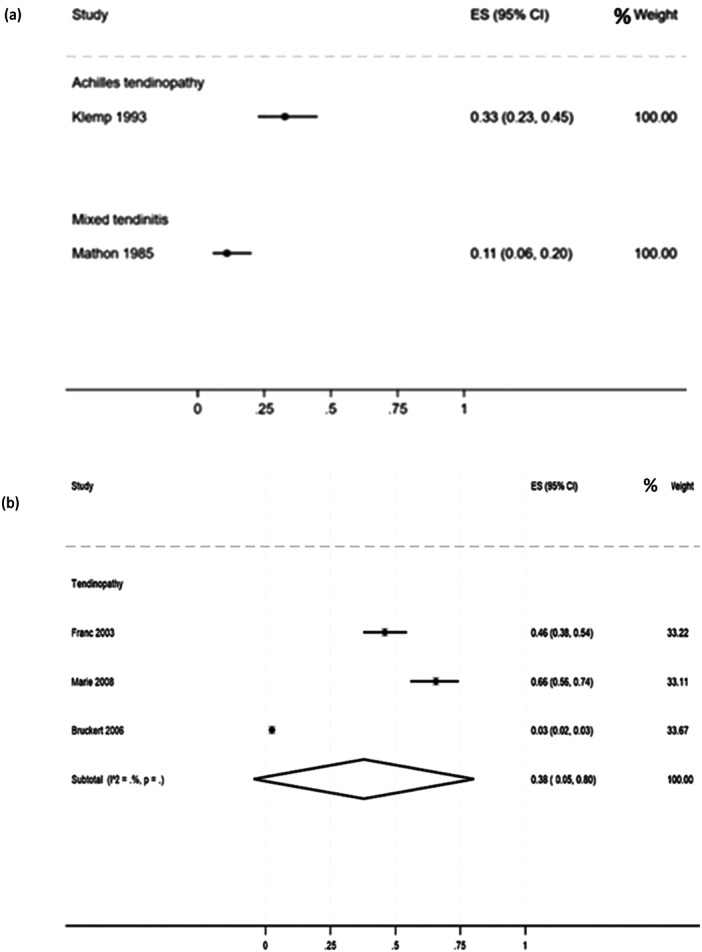
(a) Prevalence of different tendinopathies in patients with dyslipidaemia [35] and hypercholesterolaemia [45]. (b) Prevalence of different tendinopathies in patients using statins [12, 20, 43].

In other metabolic conditions, a heterogeneous prevalence of mixed types of tendinopathies was reported, ranging from 11% in hypercholesterolaemia patients (95% CI: 6%–20%, Figure 3a) [45] to 38% in hypercholesterolaemia patients using statins (95% CI: 5%–80%, Figure 3b) [12, 20, 43]. One study [35] reported Achilles' tendinopathy in patients with dyslipidaemia with a prevalence of 29% (95 CI: 15%–49%, Figure 3a).

No cross‐sectional studies reported tendinopathies in patients with alterations in BMI or MetS.

### Prevalence of metabolic alterations in patients with tendinopathies

**Figure 4:**
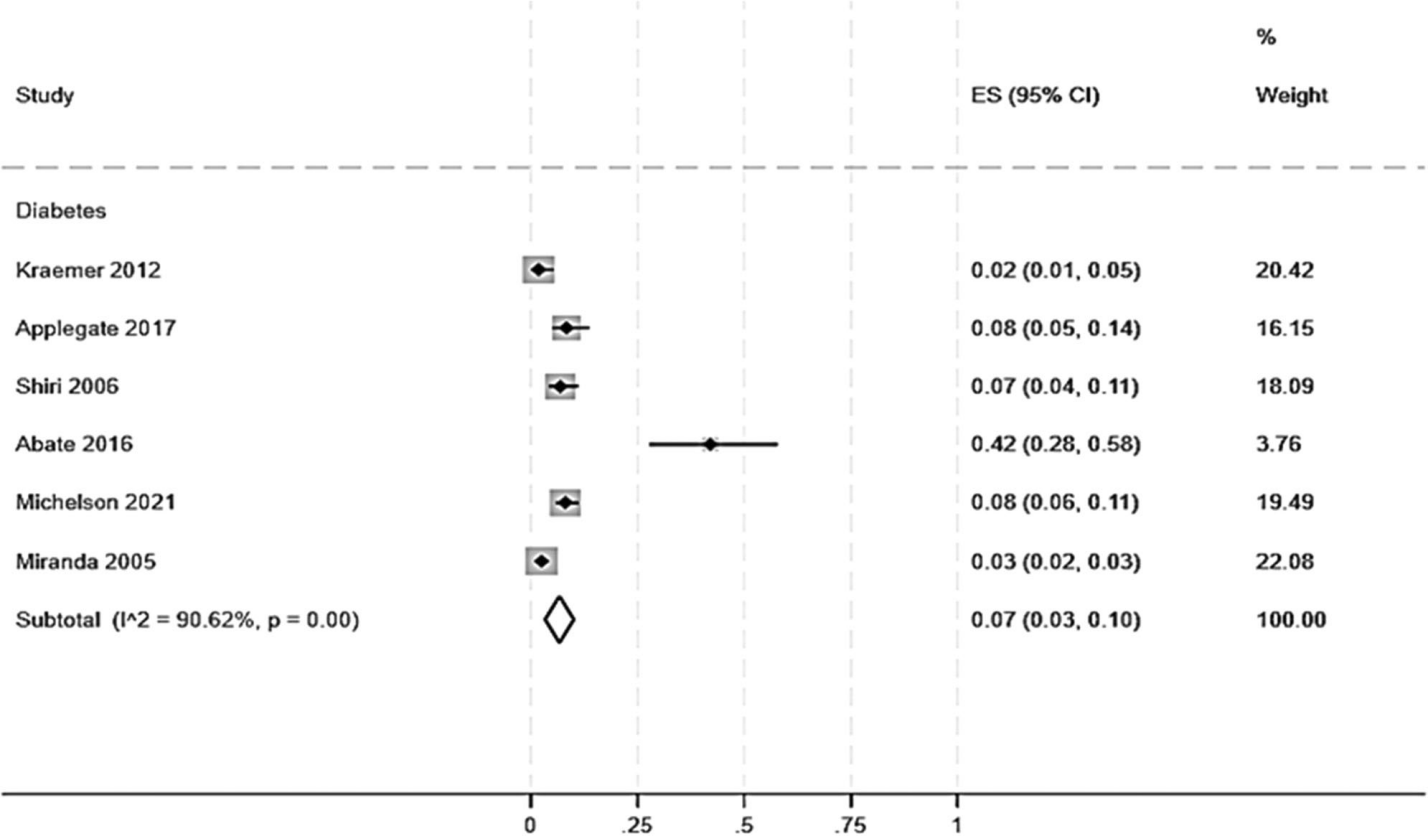
Prevalence of diabetes in patients with any kind of tendinopathy. Three cross‐sectional studies assessed the prevalence of diabetes only [3, 47, 49], two focused on diabetes and hypercholesterolaemia [8, 36], and one on diabetes and BMI alteration [62].

The studies investigating the prevalence of diabetes in patients with tendinopathies yielded an overall prevalence of 7% (95% CI: 3%–10%, Figure 4). Although Rechardt et al. met the inclusion criteria for our purpose, the data were not quantitatively analysed due to unclear outcome data.

**Figure 5:**
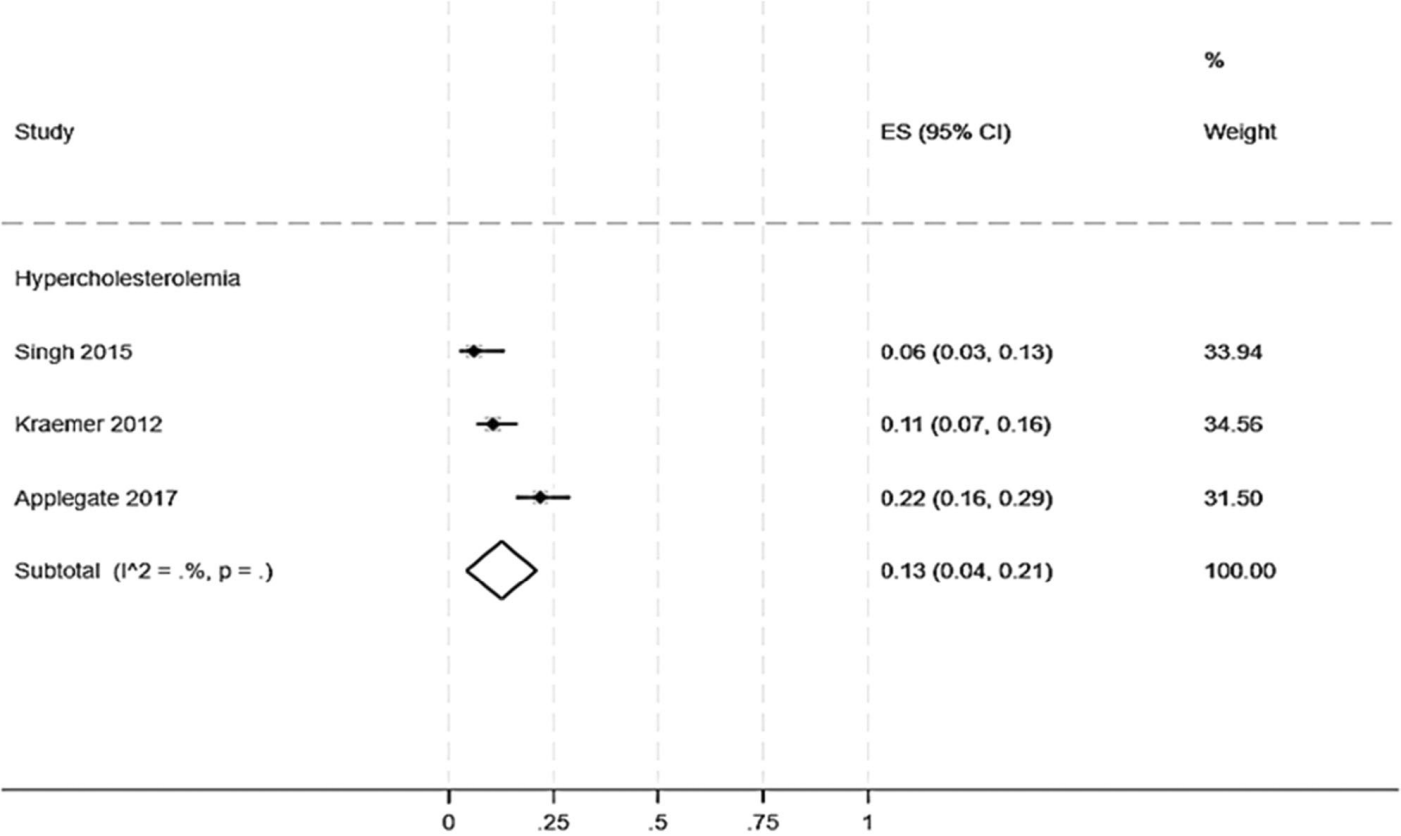
Prevalence of hypercholesterolaemia in patients with any kind of tendinopathy. One cross‐sectional study focused on hypercholesterolaemia [63], two looked at diabetes and hypercholesterolaemia [8, 36].

With regard to hypercholesterolaemia, a prevalence of 13% (95% CI: 4%–21%) was observed among patients affected by tendinopathy (Figure 5).

**Figure 6:**
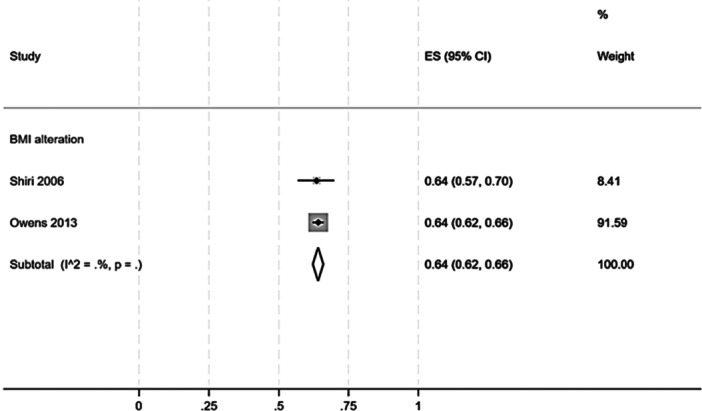
Prevalence of BMI alteration in patients with any kind of tendinopathy. One cross‐sectional study evaluated BMI alteration [55], another one diabetes and body mass index alteration [62].

The two cross‐sectional studies assessing the prevalence of BMI alterations among patients with tendinopathies reported a pooled prevalence of 64% (95% CI: 62%–66%, Figure 6).

No cross‐sectional studies were found regarding hypercholesterolaemia and statin use alterations and MetS.

### Associations between tendinopathies and metabolic alterations in studies comparing patients with and without metabolic alterations

**Figure 7:**
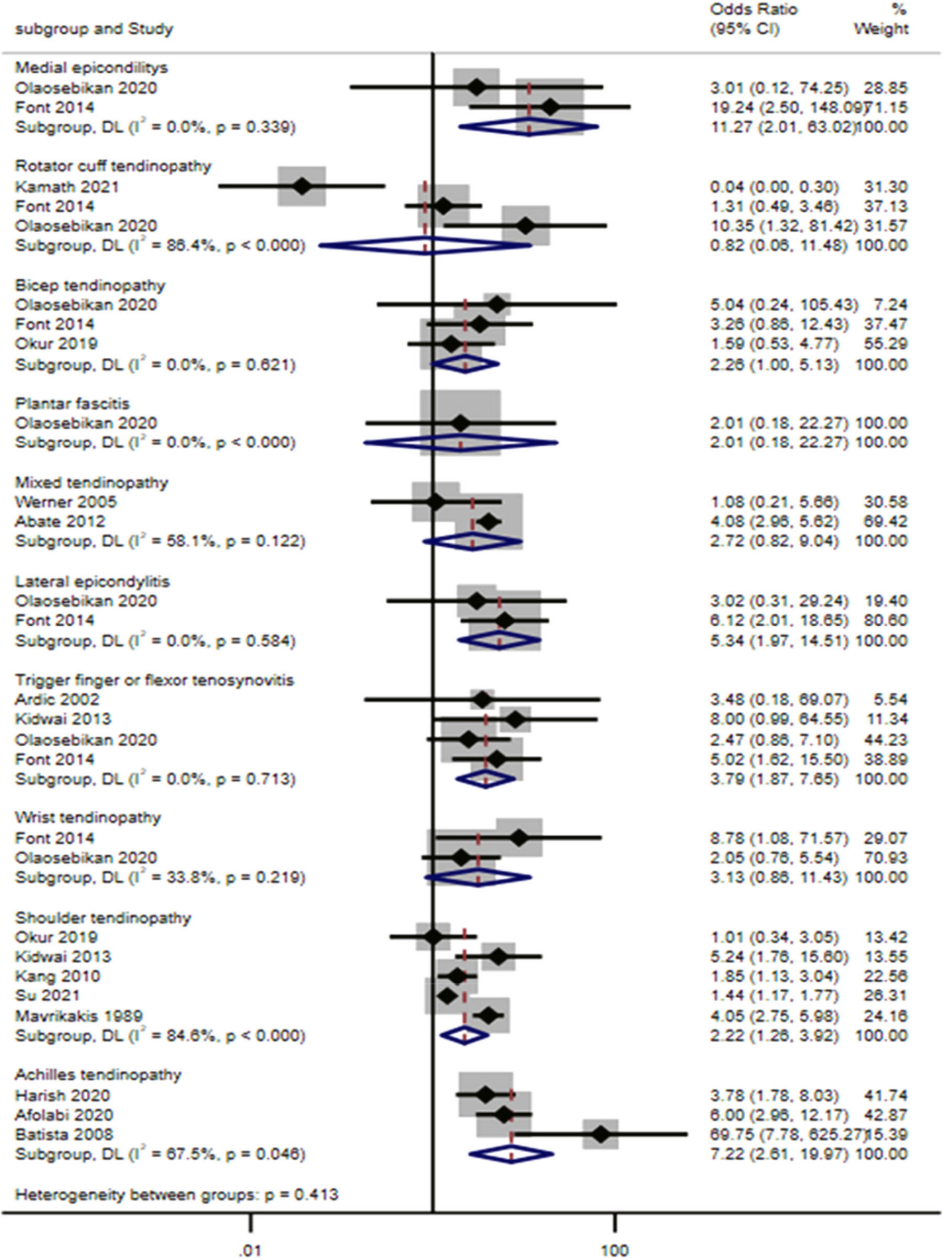
Association analysis. Forest plots showing odds ratio (OR) and 95% confidence interval (CI) of type of tendinopathy (outcome) in people with diabetes compared to people without diabetes, derived from 14 observational studies [2, 4, 9, 11, 13, 19, 22, 32, 33, 34, 46, 52, 66, 75].

Regarding the correlation between diabetes and tendinopathy, concerning lower limb tendinopathy the meta‐analysis revealed a statistically significant association between diabetes and an increased risk of Achilles tendinopathy, compared to individuals without diabetes (OR: 7.22, 95% CI: 2.61–19.97) [4, 11, 22]. One study assessed plantar fasciitis and did not show any significant association [52]. Regarding upper limb tendinopathy, the meta‐analysis revealed a statistically significant association with an increased risk in diabetic compared to nondiabetic individuals for medial epicondylitis (OR: 11.27, 95% CI: 2.01–63.02) [19, 52], lateral epicondylitis (OR: 5.34, 95% CI: 1.97–14.51) [19, 52], trigger finger or flexor tenosynovitis (OR: 3.79, 95% CI: 1.87–7.65) [9, 19, 34, 52] and rotator cuff tendinopathy (OR: 2.22, 95% CI: 1.26–3.92) [19, 32, 52] (Figure 7).

**Figure 8:**
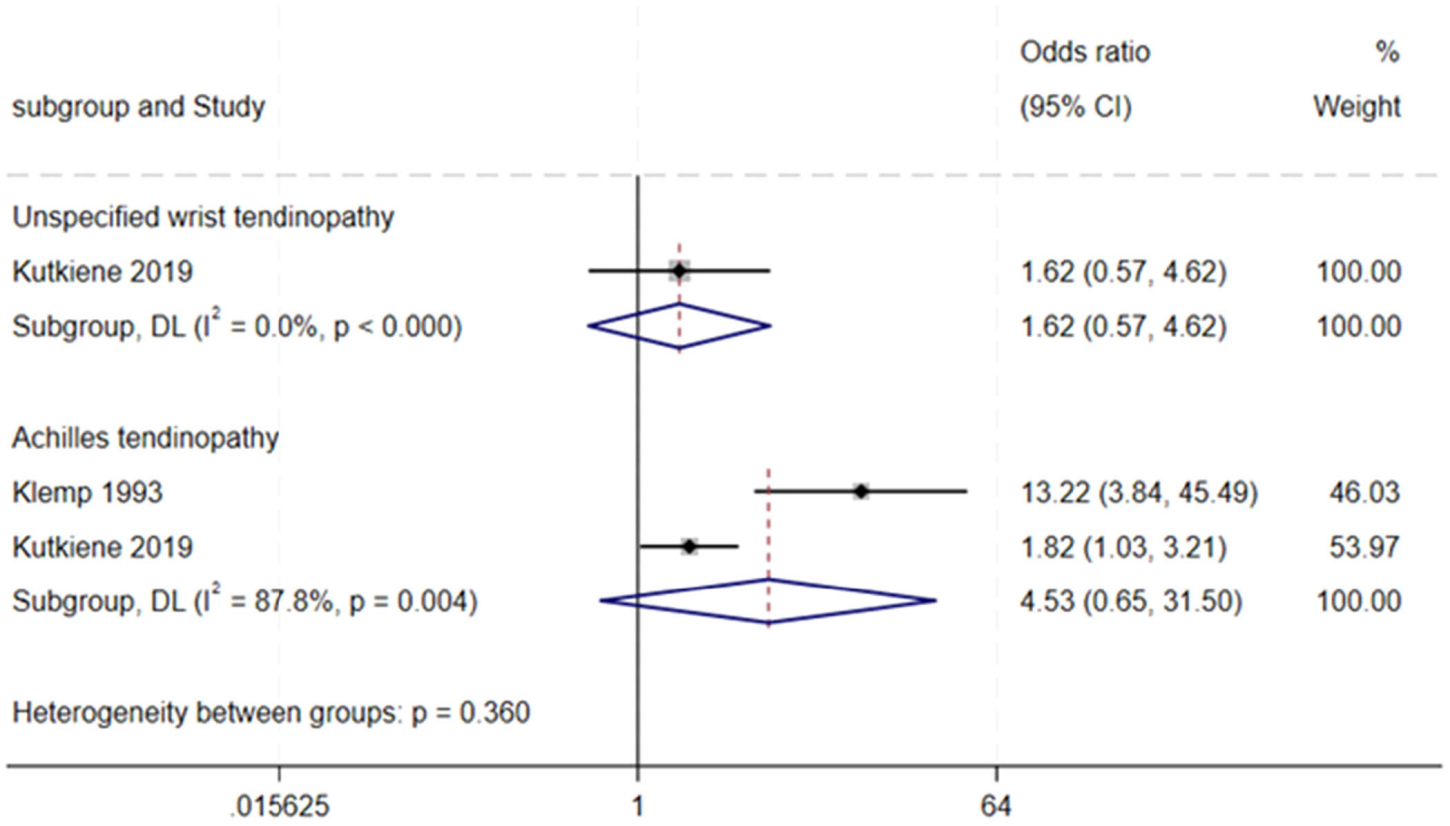
Association analysis. Forest plots showing odds ratio (OR) and 95% confidence interval (CI) of type of tendinopathy (outcome) in people with hypercholesterolaemia compared to people without hypercholesterolaemia resulting from two studies [35, 38].

In other metabolic conditions, the findings were heterogeneous. Two studies [35, 38] found no statistically significant association between tendinopathy and hypercholesterolaemia (compared to patients without hypercholesterolaemia) (Figure 8).

In contrast, a significant protective association was observed in one study [39] for hypercholesterolaemia with statin use (compared to nonusers) in the development of Achilles tendinopathy (OR: 0.54, 95% CI: 0.50–0.59), wrist tendinopathy (OR: 0.50, 95% CI: 0.46–0.50), trigger finger and tenosynovitis (OR: 0.73, 95% CI: 0.69–0.77), medial and lateral epicondylitis (OR: 0.48, 95% CI: 0.47–0.49), and shoulder tendinopathies (OR: 0.63, 95% CI: 0.61–0.64) (data not shown). One study [18] assessed enthesitis in patients with and without MetS, but the data were not analysed due to implausible estimates.

### Associations between tendinopathies and metabolic alterations in studies comparing patients with and without tendinopathies

**Figure 9:**
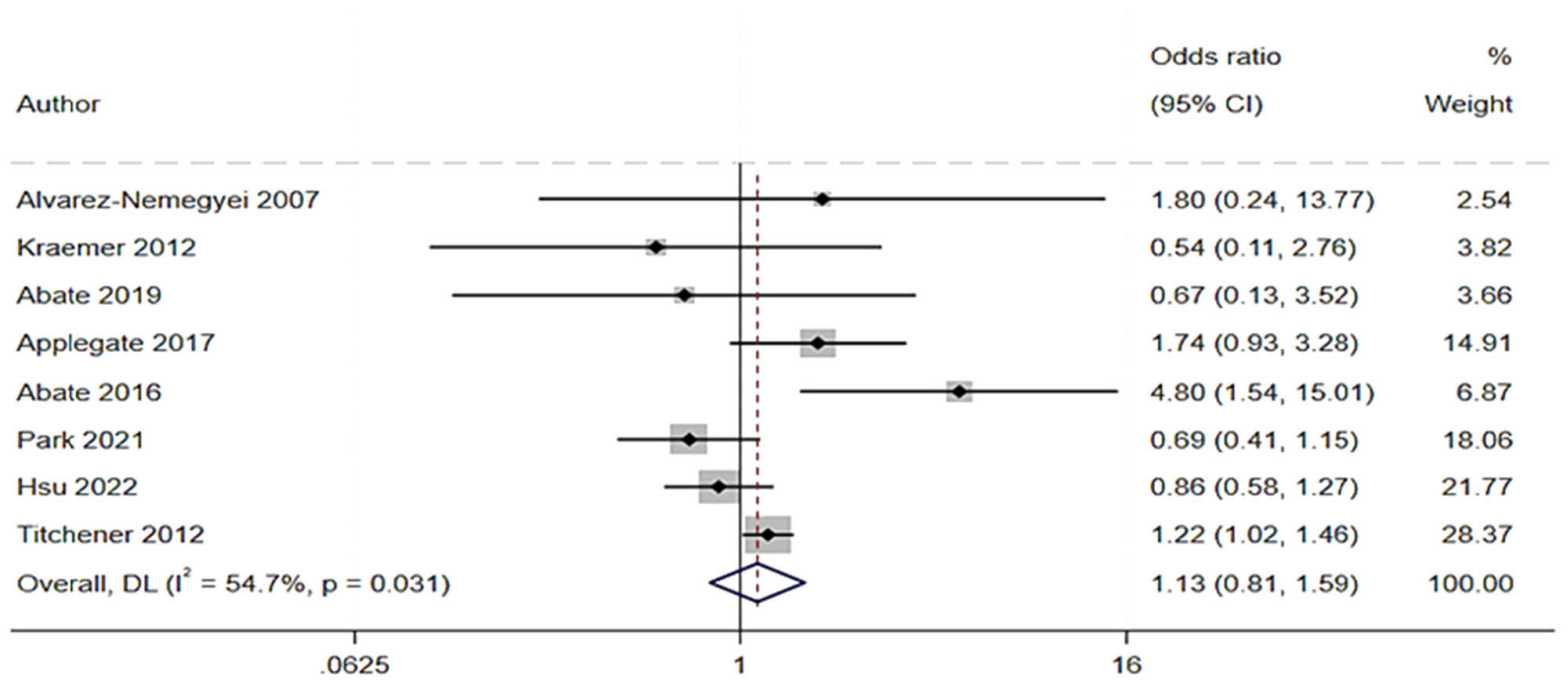
Association analysis. Forest plots showing odds ratio (OR) and 95% confidence interval (CI) of diabetes in people with tendinopathies compared to people without tendinopathies. The studies are focused on diabetes only [3], diabetes and obesity [6], diabetes and hypercholesterolaemia [8, 36], dyslipidaemia, diabetes and BMI alteration [1], dyslipidaemia and diabetes [29], diabetes, hypertriglyceridemia and metabolic syndrome [57], diabetes and BMI alteration [70].

Studies evaluating diabetes reported no significant association with tendinopathy (Figure 9).

**Figure 10:**
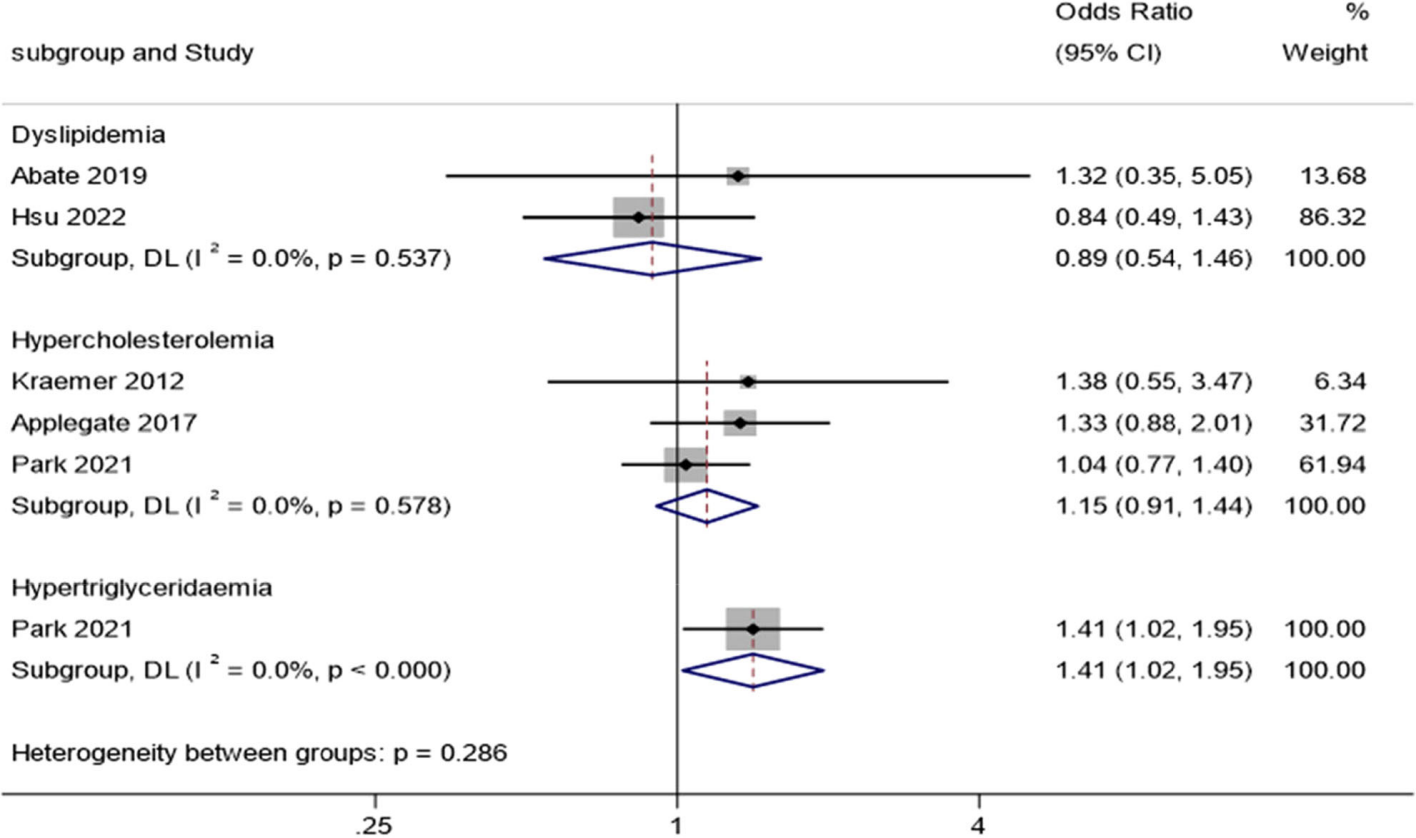
Association analysis. Forest plots showing odds ratio (OR) and 95% confidence interval (CI) of dyslipidaemia in people with tendinopathies compared to people without tendinopathies deriving from five studies [3, 8, 29, 36, 57].

Five case‐control studies [3, 8, 29, 36, 57] examining various types of dyslipidaemia disorders, including general dyslipidaemia, hypercholesterolaemia and hypertriglyceridemia revealed that tendinopathic patients had an increased likelihood of developing hypertriglyceridemia compared to healthy controls, with an OR of 1.41 (95% CI: 1.07–1.85). In contrast, the dyslipidaemia and hypercholesterolaemia categories showed a nonsignificant result (Figure 10).

**Figure 11:**
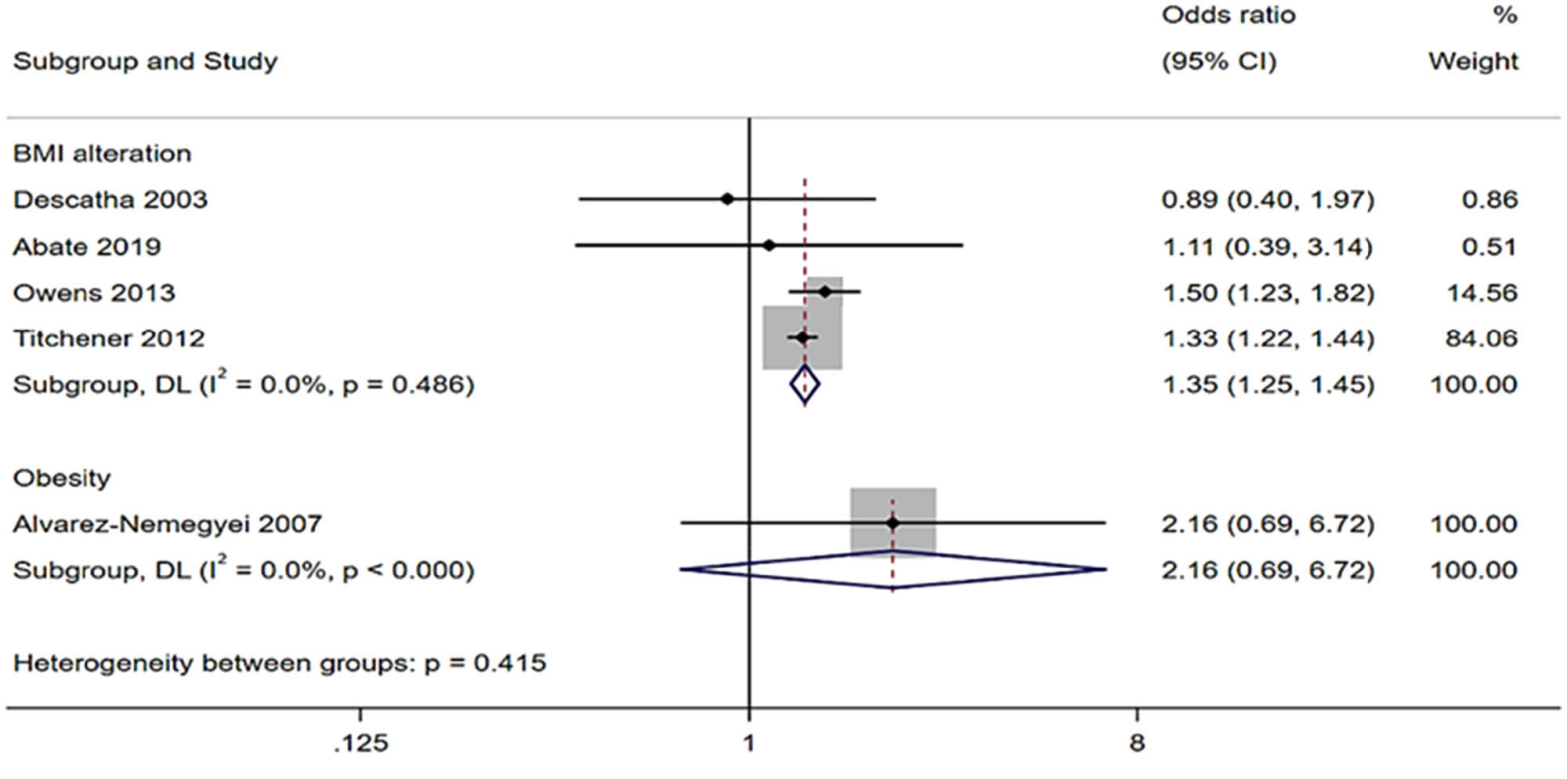
Association analysis. Forest plots showing odds ratio (OR) and 95% confidence interval (CI) of BMI alteration in people with tendinopathies compared to people without tendinopathies deriving from five case‐control studies [1, 6, 16, 55, 70].

The pooled analysis of four studies encompassing both overweight and obese participants (BMI ≥ 25 kg/m^2^), in comparison to control groups revealed an overall OR of 1.35 (95% CI: 1.25–1.45). In addition, Alvarez‐Nemegyei et al. specifically examined obesity in Pes Anserinus tendinopathy patient but did not report statistically significant findings (Figure 11). One study [57] evaluated the association with MetS but no statistically significant results was found (data not shown).

### Studies assessing sex differences

Out of the 53 studies included in the analysis, only five [6, 17, 27, 38, 59] reported different outcomes for the variable sex. Due to heterogeneity of outcome data, we narratively reported results for each single study investigating sex differences.

Particularly, Alvarez‐Nemegyei et al., included only female patients affected by Pes Anserinus tendinitis showing that no statistical difference between cases and controls was observed in the prevalence of diabetes (9.1% vs. 5.3%) and obesity (72.3% vs. 55.3%).

Rydberg et al. reported a higher prevalence of trigger finger tendinopathy in women (4%) compared to men (2.3%) in diabetic populations. Eliasson et al. evaluated the risk of trigger finger and shoulder tendinopathy among hypercholesterolemic statin users, showing an adjusted hazard ratios (aHRs) consistently higher in men (e.g., 1.50 for trigger finger, 1.43 for shoulder tendinopathy) compared to women (e.g., 1.21 for trigger finger, 1.41 for shoulder tendinopathy). Kutkiene et al. observed a markedly higher prevalence of Achilles tendon pathology in men with severe hypercholesterolaemia (60.7%) compared to women (24.1%), as well as in health individuals (55.8% vs. 2.0%, respectively). Wrist tendon pathology also showed a significant male predominance in both hypercholesterolemic (17.9% vs. 0%) and nonhypercholesterolemic populations (11.5% vs. 0%).

Holmes et al. examined the association between Achilles tendinopathy and both obesity and diabetes mellitus. The study found a statistically significant association between obesity and Achilles tendinopathy in both men and women, with a higher prevalence of obesity among women (64.3%) than men (59.5%). In contrast, diabetes mellitus was significantly associated with Achilles tendinopathy only in men younger than 44 years.

Similarly, Abate et al. analysed runners with tendinopathy and found that men with Achilles tendinopathy had a higher prevalence of diabetes (18.7%) compared to women (10%). Regarding metabolic abnormalities, BMI alterations were observed in 64.3% of women and 68.2% of men, while dyslipidaemia was identified in 30% of women versus 56.3% of men.

### Studies not included in meta‐analysis

Overall, 14 studies were not included in the meta‐analysis, of which two [18, 27] were excluded due to implausible outcome data or lack of clarity.

For the prevalence meta‐analysis, 12 studies were excluded because they did not utilise a cross‐sectional design (i.e., they were not intended to capture a ‘snapshot’ of prevalence at a single point in time) or they did not report a healthy control. However, we transparently reported raw data from these studies in Tables S1–S3.

## DISCUSSION

This systematic review and meta‐analysis provide a comprehensive synthesis of studies investigating the prevalence of tendinopathies in relation to metabolic conditions such as diabetes, dyslipidaemia, elevated BMI, and statin use in hypercholesterolaemia patients. It also examines the associations between tendinopathies and metabolic diseases. Given the multifactorial nature of tendinopathies, understanding these connections is essential for both prevention and management for individuals susceptible to tendon overload, chronic pain, or recurrent injuries, particularly in athletes and physically active populations.

### Diabetes and tendinopathy

The association of diabetes and tendinopathy has received increasing attention in recent years, with mounting evidence pointing to a robust correlation between these two conditions. Patients with diabetes have a significantly higher prevalence of tendinopathies, particularly in the lower limbs, consistent with previously studies linking diabetes to tendinous degeneration and altered collagen metabolism [72]. The prevalence of Achilles tendinopathy rates can reach up to 67% (95% CI: 42%–91%) in diabetic patients, where the meta‐analysis showed a high OR of up to 7.22 times higher compared to nondiabetic individuals, based on three studies [4, 11, 22], although two of these were of poor quality according to NOS analysis. Furthermore, the meta‐analysis reports a significant increase in the risk of upper limb tendinopathies in diabetic patients, with ORs of 2.2, 3.79, 5.34 and 11.27 for rotator cuff tendinopathy [19, 52], flexor tenosynovitis [9, 19, 34, 52], lateral epicondylitis [19, 52] and medial epicondylitis [19, 52], respectively, suggesting that repetitive stress and overuse predispose certain tendons to diabetic‐related degeneration. Several mechanisms have been hypothesised to explain this association. Chronic hyperglycaemia, which is a hallmark of diabetes, leads to the accumulation of advanced glycation end‐products (AGEs), which interfere with collagen structure, reducing tendon elasticity and making them more susceptible to degeneration and injury [68]. This is particularly evident in Achilles tendinopathy, where structural changes in the tendons have been observed [76]. In addition, microvascular damage, which is common in diabetic patients, impairs blood flow to the tendons, thereby compromising their healing ability. These factors, combined with the systemic inflammation that is frequently observed in individuals with diabetes, could contribute to the increased incidence of tendinopathy that had been observed in this group [54].

Interestingly, the overall prevalence of diabetes in patients with tendinopathies is 7% (95% CI: 3%–10%), therefore in line with the current prevalence of diabetes in the global population [28], except for a small cohort [3] which reported a 42% prevalence (95% CI: 28%–58%) and an OR of 4.80 (95% CI: 1.54–15.01).

Sex differences also seem to play a role in the prevalence and risk of tendinopathies in diabetic patients. For instance, the study by Rydberg et al. found that women with diabetes had a higher prevalence of trigger finger tendinopathy (4%) compared to men (2.3%). Similarly, within the context of Achilles tendinopathy, male subjects suffering for diabetes generally exhibit a higher prevalence than female subjects. Abate et al. further emphasised this trend, reporting a significantly higher prevalence of diabetes in men with Achilles tendinopathy (18.7%) compared to women (10%). These findings suggest that sex may influence the manifestation of tendinopathies in diabetic patients; however, the underlying reasons for these differences remain unclear.

Altogether, diabetic patients should be monitored for tendon degeneration, with particular attention to the anatomical sites most affected in line with previous studies that underscore the pivotal role of metabolic control in maintaining tendon health [10]. Early intervention encompassing physical therapy, lifestyle modifications and glycemic control has been demonstrated to reduce the incidence and severity of tendinopathies in diabetic patients [58]. However, further clinical trials are required to establish definitive causal relationships and optimal prevention strategies.

### Hypercholesterolaemia and tendinopathy in presence or absence of statin use

The relationship between hypercholesterolaemia and tendinopathy is an emerging area of interest, with a number of studies investigating their potential association. As shown in the results section, the prevalence of mixed tendinopathies in patients with hypercholesterolaemia is estimated at 11% (95% CI: 6%–20%) [45], with the Achilles tendon demonstrating the highest susceptibility, with a prevalence of 33% (95% CI: 23%–45%) [35]. It is noteworthy that this prevalence is higher in patients undergoing statin therapy, reporting a pooled prevalence of 38% (95% CI: 5%–80%) with a range from 3% to 66% [12, 20, 44]. The potential association between hypercholesterolaemia and tendinopathies is further substantiated in statin users likely attributable to an increase in metalloproteinase release following statin administration, as previously reported [17]. Nevertheless, the heterogeneity in prevalence rates highlights the need for further elucidation.

On the other hand, the data showed a pooled prevalence of 13% (95% CI: 4%–21%) [8, 36, 63] for hypercholesterolaemia in patients with tendinopathy, which was marginally higher than the prevalence observed in other metabolic conditions such as diabetes (7%), but considerably lower than BMI alterations (64%). These findings imply a potential role for BMI in the development and progression of tendinopathy.

In general, the study of association between hypercholesterolaemia and tendinopathy without the presence of statin use, has shown inconsistent results, with two studies reporting no significant data [35, 38]. A noteworthy protective association between statin use and the development of tendinopathies was indicated by Kwak et al. However, this evidence is limited to a single study and is therefore inconclusive.

In terms of sex differences, a study by Kutkiene et al. found a significantly higher prevalence of Achilles tendon pathology in men with severe hypercholesterolaemia (60.7%) compared to women (24.1%), although the underlying mechanisms for this difference remain unclear. The highest risk factor for tendinopathy, specifically of trigger finger and shoulder tendinopathy, in men compared to women is also reported when hypercholesterolemic patients are under statin use [17].

All of these findings are supported by previous clinical evidence demonstrating that elevated cholesterol levels are thought to contribute to the formation of tendon plaques, which may lead to tendon thickening and degeneration [24, 31]. Additionally, the altered lipid metabolism seen in hypercholesterolaemia may disrupt the normal turnover of collagen fibres in tendons, making them more susceptible to injury [64]. The inflammatory response associated with hypercholesterolaemia may also contribute to tendon degeneration, further increasing the risk of tendinopathies [61].

Given the potential role of cholesterol in tendon degeneration and the varying prevalence rates across studies, it is important to monitor patients with hypercholesterolaemia for signs of tendinopathy. On the other hand, clinicians should consider integrating lipid‐lowering interventions into the management of tendinopathy where appropriate.

Importantly, no studies specifically assessing the use of statin in hypercholesterolaemia patients diagnosed with tendinopathy met the inclusion criteria for this review. This finding indicates a gap in the extant literature, which hinders the ability to draw robust conclusions about the direction and magnitude of the association. To date, no definitive correlation has been demonstrated between the use of statins and the development of tendinopathy. Further studies that adequately address differences in statin dosage, length of therapy and patient comorbidities are needed to confirm any association.

### MetS and tendinopathy

MetS is a cluster of intertwined metabolic risk factors, identified by central obesity, insulin resistance, dyslipidaemia and hypertension. Three or more of these abnormalities allow the diagnosis of MetS [5]. This could be a confounding factor for our study, since the MetS includes many of the pathologic conditions we investigated individually. Therefore, it was difficult to discriminate the effect on tendinopathy resulting from one or a combination of several altered metabolic factors.

Despite this drawback, only one case‐control study [18] addressing this topic was eligible. This study reported raw data which determined an OR of 42.25 (95% CI: 14.75–121.06) for the development of enthesitis in individuals with MetS. However, these results were derived from an uneven distribution of cases and controls, raising concerns about the plausibility and reliability of the findings, therefore we excluded them from analysis.

On the other hand, regarding the evaluation of the association of MetS in tendinopathy patients, one study performed by Park et al. [57], showed that the risk of developing MetS was 15% higher in tendinopathy patients compared to the control group. Although this finding was not significant, the need for more robust and comprehensive research to confirm this observation.

These evidences underscore the importance of adopting an integrated clinical approach that addresses both tendinopathy and its metabolic contributors, while also highlighting the need for robust, longitudinal studies to validate these associations and guide evidence‐based management strategies.

### BMI alteration and tendinopathy

Despite the widespread prevalence of BMI alterations in modern populations, particularly in Western countries [67], there is an evident paucity of studies addressing their relationship with tendinopathies. This systematic review revealed a notable gap in the literature, as no eligible studies investigating the association of tendinopathies in patients with BMI alterations met our inclusion criteria. However, two cross‐sectional studies [55, 62] provided data of a pooled prevalence of 64% (95% CI: 62%–66%) of elevated BMI among patients with tendinopathies. This finding highlights the frequent coexistence of BMI alterations in tendinopathy populations. Although causality cannot be unravelled from cross‐sectional studies, the high prevalence suggests that elevated BMI may be important in the clinical evaluation and management of tendinopathies.

Furthermore, the strong relationship between tendinopathy and changes in BMI was demonstrated by four studies [1, 16, 55, 70] reporting a OR of 1.35 (95% CI: 1.25–1.45).

This association may reflect a twisted relationship: while increased mechanical loading and systemic inflammation in overweight individuals could predispose to tendinopathies [41], pain and reduced mobility associated with tendinopathies may contribute to BMI alterations over time. However, further longitudinal studies are needed to clarify the mechanisms supporting this relationship.

Interestingly, obesity, a more extreme form of BMI alteration, was considered in only one study [6]. Although the results were not statistically significant, the authors found out that obese individuals had a 116% higher risk of tendinopathy compared to those without obesity. This finding supports previous research suggesting that obesity‐related factors, such as systemic inflammation and changes in biomechanical loading, could play a role in tendon pathology [15, 41].

In terms of sex differences, Holmes et al. reported a higher prevalence of obesity in women (64.3%) compared to men (59.5%) among patients with Achilles tendinopathy. Conversely, Abate et al. observed BMI alterations in 64.3% of women and 68.2% of men with Achilles tendinopathy, suggesting unclear sex‐based differences.

On the whole, the high incidence of BMI alterations among tendinopathy patients highlights the need for incorporating weight management into tendinopathy treatment plans.

### Limitations

The first aim to assess the prevalence across all included studies at a specific time point was hampered by significant variability due to the way the data were reported in the original articles. To address this purpose, the analysis was restricted to cross‐sectional studies, ensuring more consistent data comparability. However, several limitations affect the reliability and interpretation of the findings. Risk of bias assessment revealed that the methodological quality of the included studies was highly variable, reducing the generalisability of prevalence and association estimates. The lack of high‐quality research, particularly regarding metabolic conditions, and the inconsistency in assessing associations with dyslipidaemia and MetS emphasises the necessity for standardised, prospective studies.

Additionally, insufficient data reporting addressing sex differences hindered a comprehensive analysis as our secondary outcome resulting in a narrative discussion rather than a statistical analysis. Due to heterogeneity of the pathologic conditions addressed by the included studies, the results were divided by metabolic conditions, limiting the power of analysis with small sample sizes in each subgroup. For this reason, the review was constrained by underrepresentation of certain metabolic conditions resulting in an insufficient exploration of all tendinopathy subclasses. The absence of detailed data for subgroup analysis or confounder stratification, such as age, metabolic change duration, or tendon‐specific characteristics, further restricted the quality of insights.

## CONCLUSION

The findings presented in this systematic review give a comprehensive investigation of the prevalence and association between tendinopathies and various metabolic factors, including diabetes, dyslipidaemia, BMI alterations and statin use. The results emphasised the multifactorial nature of tendinopathy pathogenesis, with metabolic conditions playing a significant role in the development of tendinopathies. Diabetes emerged as the most studied metabolic condition, showing significant associations with Achilles' tendinopathy. Other upper limb tendinopathies, also linked to diabetes, suggest that hyperglycaemia could damage tendon structure through systemic mechanisms.

Even if hypercholesterolaemia, statin use, MetS and BMI alterations are likely to be associated with tendinopathies, the literature is confined by methodological variability and a lack of longitudinal studies though evidence is limited and data inconclusive.

These findings should be a starting point for further investigation examining the metabolic factors that have been insufficiently explored to date, thereby enhancing the robustness and significance of the evidence.

## AUTHOR CONTRIBUTIONS

**Paola De Luca**: Conceptualisation; methodology; writing—original draft preparation, writing—review and editing. **Giulio Grieco**: Methodology; formal analysis and investigation; writing—original draft preparation, writing—review and editing. **Silvia Bargeri**: Formal analysis and investigation; writing—original draft preparation, writing—review and editing. **Cecilia Colombo**: Methodology; writing—original draft preparation, writing—review and editing. **Stefania Guida**: Formal analysis and investigation; writing—original draft preparation, writing—review and editing. **Michela M. Taiana**: Methodology; writing—original draft preparation, writing—review and editing. **Laura de Girolamo**: Writing—original draft preparation, writing—review and editing; funding acquisition; resources; supervision.

## CONFLICT OF INTEREST STATEMENT

The authors declare no conflict of interest.

## ETHICS STATEMENT

The study protocol was registered with the International Prospective Register of Systematic Reviews (PROSPERO) as CRD42024523183.

## Supporting information


**Supplementary table 1** Subgroups categories for data analysis.

**Supplementary table 2** Characteristics of all included studies considering tendinopathy as primary outcome in subjects with or without metabolic disease. * age range (min‐max).

**Supplementary table 3** Characteristics of all included studies considering metabolic alterations as primary outcome in subjects with or without tendinopathy. * age range (min‐max).

**Supplementary table 4** Risk of bias appraisal trough Newcastle‐Ottawa Scale (NOS) tool.

**Supplementary table 5** Risk of bias appraisal trough Quality In Prognosis Studies (QUIPS) tool.

**Supplementary Appendix 1** Query strings of different databases used for search strategy and link to repository online.

## Data Availability

All data pertinent to the study are either included in the article or provided as online supplemental information. All raw data are available at the following link: https://osf.io/bsxta/?view_only=948d3bbb5ead4ba897dd3a3801039a51.
