# Supplementary material for: The interplay between metabolic disorders and tendinopathies: Systematic review and meta‐analysis
Source: J Exp Orthop. 2025 Sep 10;12(3):e70429. doi: 10.1002/jeo2.70429 (PMC12421141; doi:10.1002/jeo2.70429)
Supplement: Supplementary file 1 — Supplementary table 1 Subgroups categories for data analysis. [file JEO2-12-e70429-s002.docx]

**Supplementary table 1**. Subgroups categories for data analysis

| **Metabolic alterations** | |
| --- | --- |
| Diabetes | Type 1 diabetes, Type 2 diabetes mellitus |
| BMI alterations | Overweight, Obesity |
| Dyslipidaemia | Hypercholesterolaemia, Hypercholesterolaemia with statin use, Hypertriglyceridaemia |
| Metabolic syndrome | / |
| **Tendinopathies** | |
| Lower limb tendinopathy | Pes Anserinus tendinopathy, Achilles tendinopathy, Plantar fasciitis |
| Upper limb tendinopathy | Shoulder tendinopathy, Rotator cuff tendinopathy, Bicep tendinopathy, Lateral and Medial Epicondylitis, De Quervain's tenosynovitis, Wrist tendinopathy, Trigger finger or flexor tenosynovitis |
