# Supplementary material for: The interplay between metabolic disorders and tendinopathies: Systematic review and meta‐analysis
Source: J Exp Orthop. 2025 Sep 10;12(3):e70429. doi: 10.1002/jeo2.70429 (PMC12421141; doi:10.1002/jeo2.70429)
Supplement: Supplementary file 2 — Supplementary table 2 Characteristics of all included studies considering tendinopathy as primary outcome in subjects with or without metabolic disease. * age range (min‐max). [file JEO2-12-e70429-s004.docx]

| **Study ID** | **Study design** | **Country** | **Funding** | **Metabolic alteration** | **Kind of tendinopathy** | **System for tendinopathy assessment** | **Study group** | **Mean age (years +/- SD)** | **Sex (%males)** | **Number of subjects (N)** |
| --- | --- | --- | --- | --- | --- | --- | --- | --- | --- | --- |
| Abate, 2012 | Case-control study (retrospective) | Italy | / | Type 2 diabetes mellitus | Multiple tendinopathy | Ultrasonography | Type 2 diabetes mellitus | 68.6 +/- 9 | 46.2% | 193 |
|  |  |  |  |  |  |  | Healthy control | 69.1 +/- 8.8 | 42.9% | 993 |
| Afolabi, 2020 | Cross-sectional study (trasversal) | Nigeria | / | Type 2 diabetes mellitus | Achilles tendinopathy | Ultrasonography | Type 2 diabetes mellitus | 60.9 +/- 10.3 | 37.5% | 80 |
|  |  |  |  |  |  |  | Healthy control | 61.0 +/- 10.3 | 42.5% | 80 |
| Ardic, 2002 | Case-control study (retrospective) | Turkey | / | Type 2 diabetes mellitus | Trigger finger | Physical examination | Type 2 diabetes mellitus | 57.8 +/- 11.9 | 29.5% | 78 |
|  |  |  |  |  |  |  | Healthy control | 55.7 +/- 11.5 | 27% | 37 |
| Batista, 2008 | Cross-sectional study (trasversal) | Brasil | / | Type 2 diabetes mellitus | Achilles tendinopathy | Ultrasonography | Type 2 diabetes mellitus | 65 +/- 13 | 41.4% | 70 |
|  |  |  |  |  |  |  | Healthy control | 67 (51-84)* | / | 10 |
| Bruckert, 2006 | Cross-sectional study (trasversal) | France | This study was supported by Novartis Pharma SAS. | Dyslipidaemia | Unspecified tendinopathy | Associated pain | Statine use | 58.4 +/- 10.8 | 65.5% | 7924 |
| Chang, 2022 | Cohort study (retrospective) | Taiwan | / | Type 2 diabetes mellitus | Shoulder tendinopathy | / | Type 2 diabetes mellitus | / | 53.9% | 63738 |
| De Carvalho Silva, 2014 | Cross-sectional study (trasversal) | Brazil | / | Type 2 diabetes mellitus | De Quervain's tenosynovitis and wrist tendinopathy | Physical examination | Type 2 diabetes mellitus | 64.9  +/- 6.4 | 4% | 100 |
|  |  |  |  | Type 2 diabetes mellitus | Trigger finger |  | Type 2 diabetes mellitus | 64.9  +/- 6.4 | 4% | 100 |
| Eliasson, 2019 | Case-control study (retrospective) | Sweden | / | Hypercholesterolemia with statine use | Achilles/Hand/Shoulder tendinopathy | / | Statine use | 69.5 +/- 8.5 | 46.5% | 37177 |
| Falsetti, 2022 | Case-control study (retrospective) | Italy | / | Metabolic syndrome | Enthesitis | Power Doppler Ultrasound | Metabolic syndrome | 60.2 +/- 8.3 | 40% | 60 |
|  |  |  |  |  |  |  | Healthy control | 61.9 +/- 7.7 | 28.3% | 60 |
| Font, 2014 | Cross-sectional study (trasversal) | Puerto Rico | supported by the National Center for Research Resources (NCRR). the National Institute on Minority Health and Health Disparities (NIMHD). and the National Center for Advancing Translational Sciences TL1TR000145 from the National Institutes of Health. and by unrestricted educational grant from Abbott Laboratories. Inc. | Type 1/Type 2 diabetes mellitus | Flexor tenosynovitis/De Quervain's tenosynovitis and wrist tendinopathy/Lateral Epicondylitis/Medial Epicondylitis/Rotator cuff tendinopathy/Bicep tendinopathy | Physical examination | Type 1/Type 2 diabetes mellitus | 53.3 +/- 12.9 | 36% | 100 |
|  |  |  |  |  |  |  | Healthy control | 50 +/- 13.1 | 35.3% | 102 |
| Franc, 2003 | Cross-sectional study (trasversal) | France | / | Dyslipidaemia | Unspecified tendinopathy | Associated pain | Statine use | 57.7 +/- 11.4 | 66.7% | 133 |
| Harish, 2020 | Cross-sectional study (trasversal) | India | / | Type 2 diabetes mellitus | Achilles tendinopathy | Sonoelastographic evaluation | Type 2 diabetes mellitus | (30-88)* | 50.6% | 154 |
|  |  |  |  |  |  |  | Healthy control | (30-75)* | 57.4% | 122 |
| Kamath, 2021 | Cross-sectional study (trasversal) | India | / | Type 2 diabetes mellitus | Shoulder tendinopathy | Ultrasonography | Type 2 diabetes mellitus | 58 (42-68)* | 43.8% | 32 |
|  |  |  |  |  |  |  | Healthy control | 54.5 (24-68)* | 40.6% | 32 |
| Kang, 2010 | Case-control study (retrospective) | Taiwan | / | Type 1/Type 2 diabetes mellitus | Shoulder tendinopathy | Ultrasonography | Type 1/Type 2 diabetes mellitus | 62.6 +/- 8.9 | 37.5% | 91 |
|  |  |  |  |  |  |  | Healthy control | 56.9 +/- 8.9 | 36.9% | 362 |
| Kidwai, 2013 | Case-control study (retrospective) | Pakistan | / | Type 2 diabetes mellitus | Shoulder tendinopathy/Trigger finger | Objective examination | Type 2 diabetes mellitus | 50.7 +/- 10.2 | 34.3% | 210 |
|  |  |  |  |  |  |  | Healthy control | 49.5 +/- 10.6 | 35% | 203 |
| Klemp, 1993 | Cross-sectional study (trasversal) | South Africa | / | Hypercholesterolemia | Achilles tendinopathy | Questionnaire, physical examination | Hypercholesterolemia | 41(19-68)* | 37% | 48 |
|  |  |  |  |  |  |  | Healthy control | 41(19-68)* | / | 48 |
| Kutkiene, 2019 | Case-control study (retrospective) | Lituania | / | Hypercholesterolemia | Achilles tendinopathy/Wrist tendinopathy | Ultrasonography | Hypercholesterolemia | 49.2 +/- 8.0 | 50.9% | 110 |
|  |  |  |  |  |  |  | Healthy control | 49.2 +/- 8.0 | 50.5% | 103 |
| Kwak, 2023 | Multiple Cohort study (prospective) | Republic of Korea | supported by the National Research Foundation of Korea funded by the Korean government (MSIT) | Hypercholesterolemia with statine use | Unspecified Tendinopathy/Trigger finger/De Quervain's tenosynovitis and wrist tendinopathy/Lateral/ Medial Epicondylitis/Shoulder tendinopathy/Achilles tendinopathy | ICD-10 system | Statine use | 49.8 +/- 12.9 | 54.4% | 84.102 |
|  |  |  |  |  |  |  | Healthy control | 50.1 +/- 14.1 | 56.2% | 168204 |
| Marie, 2008 | Cross-sectional study (trasversal) | France | / | Dyslipidaemia | Unspecified tendinopathy | Physical examination, MRI, ultrasonography and radiography occasionally | Statine use | 56 (20-89)* | 69.8% | 96 |
| Mathon, 1985 | Cross-sectional study (trasversal) | Canada | / | Hypercholesterolemia | Unspecified tendinopathy | Objective examination | Hypercholesterolemia | 35.3 +/- 16.8 | 38.4% | 73 |
| Mavrikakis, 1989 | Case-control study (retrospective) | Greece | / | Type 1/Type 2 diabetes mellitus | Shoulder tendinopathy | X rays | Type 1/Type 2 diabetes mellitus | 66.1 (36-92)* | 58.7% | 824 |
|  |  |  |  |  |  |  | Healthy control | 65.7 (34-86) * | 59.7% | 320 |
| Morén-Hybbinette, 1986 | Cross-sectional study (trasversal) | Sweden | / | Type 1/Type 2 diabetes mellitus | Shoulder tendinopathy | Associated pain, objective examination | Type 1/Type 2 diabetes mellitus | / | 63% | 62 |
| Okur, 2019 | Case-control study (retrospective) | Turkey | / | Type 2 diabetes mellitus | Shoulder tendinopathy | Ultrasonography | Type 2 diabetes mellitus | 62.4 +/- 9.6 | 38.5% | 52 |
|  |  |  |  |  |  |  | Healthy control | 66.2 +/- 7.8 | 43.5% | 46 |
| Olaosebikan, 2020 | Cross-sectional study (trasversal) | Nigeria | / | Type 2 diabetes mellitus | Trigger finger/De Quervain's tenosynovitis and wrist tendinopathy/Rotator cuff tendinopathy/Bicep tendinopathy/Lateral/ Medial Epicondylitis | Physical examination | Type 2 diabetes mellitus | 59.4 +/- 12.7 | 19.8% | 268 |
|  |  |  |  |  |  |  | Healthy control | 57.3 +/- 12.4 | 22.4% | 268 |
| Rydberg, 2022 | Cross-sectional study (trasversal) | Sweden | / | Type 1 diabetes mellitus | Trigger finger | Database | Type 1 diabetes mellitus | / | 35.8% | 9682 |
|  |  |  |  | Type 2 diabetes mellitus | Trigger finger |  | Type 2 diabetes mellitus | / | 57.7% | 85755 |
| Su, 2021 | Single Cohort study (retrospective) | Taiwan | / | Type 1/Type 2 diabetes mellitus | Shoulder tendinopathy | X-ray or ultrasound | Type 1/Type 2 diabetes mellitus | 66.9 +/- 16.1 | 50.9% | 42915 |
|  |  |  |  |  |  |  | Healthy control | 69.1   +/- 12.8 | 50.9% | 171660 |
| Unlu, 2002 | Cross-sectional study (trasversal) | Turkey | / | Type 2 diabetes mellitus | Knee tendinopathy | Ultrasonography | Type 2 diabetes mellitus | 55.1 +/- 9.3 | 29.2% | 48 |
| Werner, 2005 | Multiple Cohort study (prospective) | USA | Support for this research is provided by the National Institute on Disability and Rehabilitation Research of the United States Department of Education. Grant No. H133E980007. “Rehabilitation Engineering Research Center.” | Type 2 diabetes mellitus | Upper extremities tendinopathy | Physical examination, questionnaire | Type 2 diabetes mellitus | / | / | 7 |
|  |  |  |  |  |  |  | Healthy control | / | / | 355 |
