# Supplementary material for: The interplay between metabolic disorders and tendinopathies: Systematic review and meta‐analysis
Source: J Exp Orthop. 2025 Sep 10;12(3):e70429. doi: 10.1002/jeo2.70429 (PMC12421141; doi:10.1002/jeo2.70429)
Supplement: Supplementary file 3 — Supplementary table 3 Characteristics of all included studies considering metabolic alterations as primary outcome in subjects with or without tendinopathy. * age range (min‐max). [file JEO2-12-e70429-s001.docx]

| **Study ID** | **Study design** | **Country** | **Funding** | **Kind of tendinopathy** | **System for tendinopathy assessment** | **Metabolic alteration** | **Study group** | **Mean age (years +/- SD)** | **Sex (% males)** | **Number of subjects (N)** |
| --- | --- | --- | --- | --- | --- | --- | --- | --- | --- | --- |
| Abate, 2016 | Cross-sectional study (trasversal) | Italy | / | Achilles tendinopathy | Ultrasonography | Type 2 diabetes mellitus | Achilles tendinopathy | 69.6 +/- 3.3 | 84% | 38 |
|  |  |  |  |  |  |  | Healthy control | 69 +/- 2.8 | 84% | 38 |
| Abate, 2019 | Case-control study (retrospective) | Italy | / | Achilles tendinopathy | Ultrasonography | Type1 and Type 2 diabetes mellitus/Dyslipidaemia | Achilles tendinopathy | / | 62% | 26 |
|  |  |  |  | Achilles tendinopathy |  | BMI alteration | Achilles tendinopathy | 39.3 +/- 12.9 | 61% | 36 |
|  |  |  |  |  |  | BMI alteration | Healthy control | 39.1 +/- 7.6 | 64% | 28 |
| Alvarez-Nemegyei, 2007 | Case-control study (retrospective) | Mexico | / | Knee tendinopathy | Physical examination | Type 1/Type 2 diabetes mellitus/BMI alteration | Pes Anserinus tendinopathy /Bursitis | 62.1 +/- 11.5 | 0% | 22 |
|  |  |  |  |  |  |  | Healthy control | 59.8 +/- 9.4 | 0% | 38 |
| Applegate, 2017 | Cross-sectional study (trasversal) | USA | / | Rotator cuff tendinopathy | Physical examination | Type 2 diabetes mellitus/Hypercholesterolemia | Rotator cuff tendinopathy | 45.6 +/- 10.7 | 28.2% | 156 |
|  |  |  |  |  |  |  | Healthy control | 41.6 +/- 11.4 | 35.2% | 1070 |
| Blyth, 1996 | Case-control study (retrospective) | England | / | Hand tendinopathy | / | Type 1/Type 2 diabetes mellitus | Trigger Finger | / | 33.3% | 100 |
| Descatha, 2003 | Single Cohort study (prospective) | France | INSERM | Epicondilytis | Physical examination | BMI alteration | Epicondilytis | / | 26% | 68 |
|  |  |  |  |  |  |  | Healthy control | / | 25% | 1689 |
| Fernández Cuadros, 2020 | Single Cohort study (prospective) | Spain | / | Shoulder tendinopathy | Radiological or ultrasound diagnosis | Type 2 diabetes mellitus | Shoulder tendinopathy | 54.6 | 26.9% | 138 |
| Gilotra, 2021 | Case-control study (retrospective) | USA | / | Elbow tendinopathy | MRI | Type 2 diabetes mellitus | Extensor carpi radialis brevis tendinopathy | 47.9 +/- 7.9 | 58% | 12 |
| Holmes, 2006 | Case-control study (retrospective) | USA | / | Achilles tendinopathy | Radiographs, MRI | BMI alteration | Achilles tendinopathy | 50.5 ( 27 -77)* | 46% | 79 |
|  |  |  |  | Achilles tendinopathy |  | Type 1/Type 2 diabetes mellitus | Achilles tendinopathy | 50.5 ( 27 -77)* | 47% | 82 |
| Hsu, 2022 | Case-control study (retrospective) | Taipei | Taipei Medical University–Shuang Ho Hospital (110TMU- SHH-14) | Shoulder tendinopathy | Ultrasound, physical examination | Type 2 diabetes mellitus/Dyslipidaemia | Shoulder tendinopathy | 57.1 +/- 12.3 | 48% | 308 |
|  |  |  |  |  |  |  | Healthy control | 57.2 +/- 13.0 | 47.7% | 300 |
| Kraemer, 2012 | Cross-sectional study (trasversal) | Germany | / | Achilles tendinopathy | Questionnaire | Type 1/Type 2 diabetes mellitus/Hypercholesterolemia | Achilles tendinopathy | 41 +/- 11 | 68% | 161 |
|  |  |  |  |  |  |  | Healthy control | 39 +/- 11 | 64% | 89 |
| Lagas, 2020 | Single Cohort study (prospective) | Netherlands | financial support from the Netherlands Organization for Health Research and Development (ZonMw) | Achilles tendinopathy | Questionnaire | Type 1/Type 2 diabetes mellitus/Hypercholesterolemia | Achilles tendinopathy | 47.1 +/- 10 | 69% | 62 |
| Martin, 2019 | Single Cohort study (prospective) | Spain | grant ISCIII (PI13/01707) cofinanced by FEDER funds | Elbow tendinopathy | Pain assessment | Type 1/Type 2 diabetes mellitus/Hypercholesterolemia | Elbow tendinopathy | 49.5  +/- 7.16 | 46.5% | 71 |
| Michelson, 2021 | Single Cohort study (retrospective) | USA | / | Foot tendinopathy | Presence of tenderness and/or pain localized to the FHL | Type 2 diabetes mellitus | Tendinopathy | 45 +/- 15 | 26% | 409 |
| Miranda, 2005 | Cross-sectional study (trasversal) | Finland | / | Shoulder tendinopathy | Physical examination | Type 1/Type 2 diabetes mellitus | Rotator cuff tendinopathy | (30-64)* | 51% | 3885 |
| Neto, 2021 | Single Cohort study (retrospective) | Brazil | / | Trigger finger | Physical examination | Metabolic syndrome | Trigger finger | 63 (50-84)* | 32% | 75 |
| Owens, 2013 | Cross-sectional study (trasversal) | USA | / | Achilles tendinopathy | / | BMI alteration | Achilles tendinopathy | / | 69.3% | 450 |
|  |  |  |  | Knee tendinopathy |  | BMI alteration | Knee tendinopathy | / | 63.5% | 584 |
|  |  |  |  | Foot tendinopathy |  | BMI alteration | Foot tendinopathy | / | 56.6% | 1228 |
|  |  |  |  |  |  | BMI alteration | Healthy control | / | 70.3% | 77902 |
| Park, 2021 | Case-control study (retrospective) | Republic of Korea | / | Epicondylitis | Ultrasonography, MRI | Type 1/Type 2 diabetes mellitus/Hypercholesterolemia/Hypertriglyceridaemia | Lateral Epicondylitis | 58.8 +/- 8.9 | 32.7% | 245 |
|  |  |  |  |  |  |  | Healthy control | 60 +/- 8.5 | 53.3% | 692 |
| Ptak, 2023 | Single Cohort study (retrospective) | USA | / | Achilles tendinopathy | / | BMI alteration | Unspecified tendinopathy | 58.9 +/- 11.4 | 34.6% | 78 |
| Rechardt, 2010 | Cross-sectional study (trasversal) | Finland | / | Shoulder tendinopathy | Pain assessment, physical examination | Type 1 diabetes | Rotator cuff tendinopathy | / | 45.8% | 35 |
|  |  |  |  |  |  | Type 2 diabetes mellitus | Rotator cuff tendinopathy | / | 45.8% | 318 |
|  |  |  |  |  |  | BMI alteration | Rotator cuff tendinopathy | / | 45.8% | 3255 |
|  |  |  |  |  |  | Metabolic syndrome | Rotator cuff tendinopathy | / | 45.8% | 1884 |
| Roh, 2017 | Case-control study (retrospective) | Republic of Korea | / | Lateral Epicondylitis | Questionnaire, phisical examination | Type 1/Type 2 diabetes mellitus/ Hypertriglyceridaemia | Lateral Epicondylitis | 45.95 (25-60)* | 35.3% | 102 |
| Schon, 2013 | Single Cohort study (prospective) | USA | / | Achilles tendinopathy | Clinical, radiographic evaluation | Type 2 diabetes mellitus | Achilles tendinopathy | 54 +/- 10 | / | 46 |
| Shiri, 2006 | Cross-sectional study (trasversal) | Finland | / | Epicondylitis | Interview, physical examination | BMI alteration | Lateral Epicondylitis | / | / | 126 |
|  |  |  |  |  |  | Type 1/Type 2 diabetes mellitus | Lateral Epicondylitis | / | / | 131 |
|  |  |  |  |  |  | BMI alteration | Medial Epicondylitis | / | / | 83 |
|  |  |  |  |  |  | Type 1/Type 2 diabetes mellitus | Medial Epicondylitis | / | / | 86 |
| Singh, 2015 | Cross-sectional study (trasversal) | UK | / | Achilles tendinopathy | Clinical diagnosis | Hypercholesterolemia | Achilles tendinopathy | / | 68.7% | 83 |
| Titchener, 2012 | Case-control study (retrospective) | UK | / | Epicondilytis | Database | Type 1/Type 2 diabetes mellitus/ BMI alteration | Lateral epicondylitis | / | / | 4998 |
|  |  |  |  |  |  |  | Healthy control | / | / | 4998 |
