# Supplementary material for: The interplay between metabolic disorders and tendinopathies: Systematic review and meta‐analysis
Source: J Exp Orthop. 2025 Sep 10;12(3):e70429. doi: 10.1002/jeo2.70429 (PMC12421141; doi:10.1002/jeo2.70429)
Supplement: Supplementary file 4 — Supplementary table 4 Risk of bias appraisal trough Newcastle‐Ottawa Scale (NOS) tool. [file JEO2-12-e70429-s003.pdf]

Cross-sectional studies

	1.Selection													SELECTION	2.Comparability		COMPARABILITY	3.Outcome						OUTCOME	Overall quality score (stars)	Overall JUDGMENT
	1. Representativeness of the sample				2. Sample size			3. Non-respondents			4) Ascertainment of the exposure (risk factor) :			score	1) The subjects in different outcome groups are comparable, based on the study design or analysis. Confounding factors are controlled.		score	1) Assessment of the outcome				2) Statistical test:		score		
First author, year	a) Truly representative of the average in the traget population * (all subject or random sampling)	b) somewhat representative of the average in the target population * (non-random sampling)	c) selected group of users	d) no description of the sampling strategy.	a) Justified and satisfactory *	b) Not justified	c) no informations	a) Comparability between respondents and non-respondents characteristics is established, and the response rate is satisfactory. *	b) The response rate is unsatisfactory, or the comparability between respondents and non-respondents is unsatisfactory.	c) No description of the response rate or the characteristics of the responders and the non-responders.	a)Vaccine records/vaccine registry/clinic registers/ hospital records only. **	b)Parental or personal recall and vaccine/hospital records..*	c)Parental/personal recall only.		Data/ results adjusted for relevant predictors /risk factors/confounders e.g. age, sex, time since vaccination, etc.a) The study controls for the most important factor (select one). **	Data/results not adjusted for all relevant confounders/risk factors/information not provided.		a)Independent blind assessment using objective validated laboratory methods **	b)Unblinded assessment using objective validated laboratory methods. **	c)Used non-standard or non-validated laboratory methods with gold standard. *	d)No description/non-standard laboratory methods used.	a) The statistical test used to analyze the data is clearly described and appropriate, and the measurement of the association is presented, including confidence intervals and the probability level (p value). *	b) The statistical test is not appropriate, not described or incomplete			
**MET**																										
De Carvalho E Silva, 2014			X			X				X	X			2*		X	0*			X			X	1*	3*	poor
Font, 2014		X				X				X	X			3*	X		2*			X		X		2*	7*	fair
Batista, 2008		X				X				X	X			3*		X	0*		X				X	2*	5*	poor
Afolabi,2020		X				X				X	X			3*	X		2*		X				X	2*	7*	good
Harish,2020			X			X				X		X		1*		X	0*		X			X		3*	4*	poor
Olaosebikan, 2020		X			X					X	X			3*		X	0*		X				X	2*	6*	poor
Klemp, 1993		X				X				X		X		2*	X		2*				X		X	0*	4*	poor
Franc, 2003		X				X				X		X		2*		X	0*				X		X	0*	2*	poor
Mathon, 1985		X				X				X		X		2*		X	0*				X		X	0*	2*	poor
Unlu, 2002			X				X			X	X			2*		X	0*		X				X	2*	4*	poor
Kamath, 2021			X		X			X			X			4*		X	0*			X			X	1*	5*	poor
Bruckert, 2006		X				X				X	X			3*	X		2*			X		X		1*	7*	poor
Marie, 2008	X					X				X		X		2*		X	0*		X				X	2*	4*	poor
Moren-Hybbinette, 1986			X			X				X	X			2*		X	0*		X				X	2*	4*	poor
Rydberg, 2022	X					X				X	X			3*	NA					X		X		2*	5*	good
**TEND**																										
Singh, 2015			X			X				X	X			2*		X	0*		X				X	2*	4*	poor
Michelson, 2021			X			X		X			X			3*		X	0*				X	X		3*	4*	poor
Owens, 2013		X				X				X	X			3*		X	0*			X		X		2*	6*	poor
Kraemer, 2012			X			X				X			X	0*	X		2*				X	X		1*	3*	poor
Rechardt, 2010	X				X					X		X		3*	X		2*		X			X		3*	8*	good
Shiri, 2006	X				X					X		X		3*	X		2*		X			X		3*	8*	good
Abate, 2016			X			X				X	X			2*	X		2*			X			X	1*	5*	poor
Applegate, 2017	X				X				X			X		3*	X		2*		X			X		3*	8*	good
Miranda, 2005	X				X					X		X		3*	X		2*			X		X		2*	7*	good

Case-control studies

First author, year	1.Selection										SELECTION	2.Comparability		COMPARABILITY	3.Exposure										EXPOSURE	Overall quality score (stars)	Overall JUDGMENT
	1. Is the case definition adequate			2. Representativeness of the cases		3. Selection of Controls			4) Definition of Controls		score	1) Comparability of cases and controls on the basis of the design or analysis		score	1) Assessment of the exposure					2) Same method of ascertainment for cases and controls		3) Non-Response rate			score		
	a)yes, with independent validation *	b) yes, eg record linkage or based on self reports *	c) no description	a) consecutive or obviously representative series of cases *	b) potential for selection biases or not stated	a) community controls *	b) hospital controls	c) no description	a) no history of disease (endpoint) *	b) no description of source		a) study controls for ______ (Select the most important factor.) *	b) study controls for any additional factor * (This criteria could be modified to indicate specific control for a second important factor.)		a) secure record (eg surgical records) *	b) structured interview where blind to case/control status *	c) interview not blinded to case/control status	d) written self report or medical record only	e) no description	a) yes *	b) no	a)same rate for both groups *	b) non respondents described	c) rate different and no designation			
**MET**																											
Abate, 2012	X				X		X			X	1*	NA	NA		X					X				X	2*	3*	poor
Kutkiene, 2019	X				X		X			X	1*	X		1*	X					X				X	2*	5*	poor
Eliasson, 2019		X		X		X			X		3*	X		1*	X					X				X	2*	7*	poor
Kidwai, 2013		X		X			X		X		3*	X		1*	X					X				X	2*	6*	good
Mavrikakis,1989	X			X			X			X	2*	X		1*	X					X				X	2*	5*	fair
Okur, 2019	X				X		X			X	1*	X		1*	X					X				X	2*	4*	poor
Kang, 2010		X		X			X			X	2*	X		1*			X			X				X	1*	4*	poor
Ardic, 2003		X		X			X			X	2*	X		1*	X					X				X	2*	5*	fair
Falsetti,2022		X		X			X			X	2*	X		1*	X					X				X	2*	5*	fair
**TEND**																											
Hsu, 2022	X				X	X				X	2*	X		1*	X					X				X	2*	5*	fair
Abate, 2019	X				X	X				X	2*	X		1*	X					X				X	2*	5*	fair
Alvarez-Nemegyei, 2007	X			X			X			X	2*	X		1*			X			X				X	1*	4*	poor
Park, 2021	X			X		X				X	3*	NA	NA		X					X				X	2*	5*	good
Holmes, 2006	X			X			X			X	2*	X		1*	X					X				X	2*	5*	fair
Roh, 2017		X		X		X				X	3*	X		1*			X			X				X	1*	4*	poor
Titchener, 2012		X		X		X				X	2*	X		1*	X					X				X	2*	6*	fair
Gilotra, 2021	X				X		X			X	1*	X		1*	X					X				X	2*	4*	poor
Blyth, 1996		X		X			X			X	2*	NA	NA		X					X				X	2*	4*	fair

Cohort studies

First author, year	1.Selection														SELECTION	2.Comparability		COMPARABILITY	3.Outcome										OUTCOME	Overall quality score (stars)	Overall JUDGMENT
	1. Representativeness of the exposed cohort				2. Selection of the non exposed cohort			3. Ascertainment of exposure				4) Demonstration that outcome of interest was not present at start of study		score	1) Comparability of cohorts on the basis of the design or analysis		score	1) Assessment of the outcome				2) Was follow-up long enough for outcomes to occur		3) Adequacy of follow up of cohorts				score		
	a)truly representative of the average _______________ (describe) in the community *	b) somewhat representative of the average ______________ in the community *	c) selected group of users eg nurses, volunteers	d) no description of the derivation of the cohort	a) drawn from the same community as the exposed cohort *	b) drawn from a different source	c) no description of the derivation of the non exposed cohort	a) secure record (eg surgical records) *	b) structured interview *	c)written self report	d)no description	a) yes *	b) no		a) The study control for any additional factor. *	b) study controls for any additional factor * (This criteria could be modified to indicate specific control for a second important factor.)		a) independent blind assessment **	b) record linkage **	c) self report	d) no description	a) yes (select an adequate follow up period for outcome of interest) *	b) no	a) complete follow up - all subjects accounted for *	b) subjects lost to follow up unlikely to introduce bias * small number lost - > ____ % (select an adequate %) follow up, or description provided of those lost) *	follow up rate < ____% (select an adequate %) and no description of those lost	no statement			
**MET**																															
Chang, 2022	X				X			X				X		4*	X		1*		X			X		X				4*	9*	fair
Su, 2021	X				X			X				X		4*	X		1*		X			X		X				4*	9*	fair
Werner, 2005	X				X				X			X		4*	X		1*		X			X			X			4*	9*	fair
**TEND**																															
Ptak, 2024		X			single cohort study			X				X		3*	NA				X			X		X				4*	7*	good
Martin, 2019		X			single cohort study			X				X		3*	NA				X			X					X	3*	6*	good
Fernández Cuadros, 2020		X			single cohort study			X				X		3*	NA				X			X		X				4*	7*	good
Lagas, 2020			X		single cohort study					X			X	0*	NA					X		X			X			2*	2*	poor
Neto JHS, 2021			X		single cohort study			X				X		2*	NA				X			X					X	3*	5*	fair
Schon, 2013			X					X				X		3*	NA				X			X				X		3*	5*	good
Descatha, 2003		X			X				X			X		4*	NA					X		X			X			2*	6*	good
