# Supplementary material for: The interplay between metabolic disorders and tendinopathies: Systematic review and meta‐analysis
Source: J Exp Orthop. 2025 Sep 10;12(3):e70429. doi: 10.1002/jeo2.70429 (PMC12421141; doi:10.1002/jeo2.70429)
Supplement: Supplementary file 5 — Supplementary table 5 Risk of bias appraisal trough Quality In Prognosis Studies (QUIPS) tool. [file JEO2-12-e70429-s005.pdf]

***Prognostic studies***

| First author, year | 1. Study Participation | | | | | | | | | | Summary Study Participation | | 2. Study Attrition | | | | | | | | | | Summary Study Attrition | |
|---|---|---|---|---|---|---|---|---|---|---|---|---|---|---|---|---|---|---|---|---|---|---|---|---|
| | The source population or population of interest is adequately described for key characteristics | | The sampling frame and recruitment are adequately described, possibly including methods to identify the sample, place of recruitment, and period of recruitment | | Inclusion and exclusion criteria are adequately described | | There is adequate participation in the study by eligible individuals | | The baseline study sample is adequately described for key characteristics | | The study sample represents the population of interest on key characteristics, sufficient to limit potential bias of the observed relationship between the prognostic factor and outcome | | Response rate is adequate and is > 80% | | Attempts to collect information on participants who dropped out of the study are described | | Reasons for loss to follow up are described | | Participants lost to follow up are adequately described for key characteristics | | There are no important differences between key characteristics and outcomes in participants who completed the study and those who did not | | Loss to follow-up is not associated with key characteristics sufficient to limit potential bias to the observed relationship between the prognostic factor and the outcome | |
| | **Yes** | **No** | **Yes** | **No** | **Yes** | **No** | **Yes** | **No** | **Yes** | **No** | **Yes** | **No** | **Yes** | **No** | **Yes** | **No** | **Yes** | **No** | **Yes** | **No** | **Yes** | **No** | **Yes** | **No** |
| **MET** | | | | | | | | | | | | | | | | | | | | | | | | |
| Kwak, 2023 | X | | X | | X | | X | | X | | X | | X | | | X | | X | | X | | X | | X |

| | 3. Prognostic Factor Measurement | | | | | | | | | | | | | | PF Measurement Summary | | 4. Outcome Measurement | | | | | | Outcome Measurement Summary | |
|---|---|---|---|---|---|---|---|---|---|---|---|---|---|---|---|---|---|---|---|---|---|---|---|---|
| | A clear definition or description of the prognostic factors is provided | | Method of prognostic factor measurement is adequately valid and reliable to limit misclassification bias | | The prognostic factors measured are blinded for outcome measure | | Continuous variables are reported or appropriate cut-offs are used | | The method and setting of measurement of PF is the same for all study participants | | More than 80% of the study sample has completed data for PF variable | | Appropriate methods of imputation are used for missing 'PF' data | | PF is adequately measured in study participants to sufficiently limit potential bias | | A clear definition of the Outcome is provided | | The method of outcome measurement used in valid and reliable to limit misclassification bias | | The method and setting of outcome measurement is the same for all study participants | | Outcome of interest is adequately measured in study participants to sufficiently limit potential bias | |
| | **Yes** | **No** | **Yes** | **No** | **Yes** | **No** | **Yes** | **No** | **Yes** | **No** | **Yes** | **No** | **Yes** | **No** | **Yes** | **No** | **Yes** | **No** | **Yes** | **No** | **Yes** | **No** | **Yes** | **No** |
| **MET** | | | | | | | | | | | | | | | | | | | | | | | | |
| Kwak, 2023 | X | | X | | | X | | X | X | | X | | | X | | X | X | | X | | X | | X | |

| | 5. Study Confounding | | | | | | | | | | Study Confounding Summary | | 6. Statistical Analysis and Reporting | | | | | | | | | | | | Statistical Analysis and Reporting Summary | | |
|---|---|---|---|---|---|---|---|---|---|---|---|---|---|---|---|---|---|---|---|---|---|---|---|---|---|---|---|
| | All important confounders are measured | | Clear definitions of the important confounders measured are provided | | The method and setting of confounding measurement are the same for all study participants | | Important potential confounders are accounted for in the study design | | Important potential confounders are accounted for in the analysis | | Important potential confounders are appropriately accounted for, limiting potential bias with respect to the relationship between PF and outcome | | There is sufficient presentation of data to assess the adequacy of the analysis | | The strategy for model building is appropriate and is based on a conceptual framework or model. | | The selected statistical model is adequate for the design of the study | | There is a description of the association of the prognostic factor and the outcome, including information about the statistical significance | | Continuous variables are reported or cut-off points are used | | There is no selective reporting of results | | The statistical analysis is appropriate for the design of the study, limiting potential for presentation of invalid or spurious results | | **overall JUDGEMENT** |
| | **Yes** | **No** | **Yes** | **No** | **Yes** | **No** | **Yes** | **No** | **Yes** | **No** | **Yes** | **No** | **Yes** | **No** | **Yes** | **No** | **Yes** | **No** | **Yes** | **No** | **Yes** | **No** | **Yes** | **No** | **Yes** | **No** | |
| **MET** | | | | | | | | | | | | | | | | | | | | | | | | | | | |
| Kwak, 2023 | X | | X | | | X | | X | X | | X | | X | | X | | X | | X | | | X | | X | X | | Low |
