# Supplementary material for: The interplay between metabolic disorders and tendinopathies: Systematic review and meta‐analysis
Source: J Exp Orthop. 2025 Sep 10;12(3):e70429. doi: 10.1002/jeo2.70429 (PMC12421141; doi:10.1002/jeo2.70429)
Supplement: Supplementary file 6 — Supplementary Appendix 1 Query strings of different databases used for search strategy and link to repository online. [file JEO2-12-e70429-s006.docx]

**Supplementary Appendix 1**

Search strings for each browser:

Pubmed: (263 results)

("Metabolic Diseases"[Title/Abstract] OR "Metabolic Alterations"[Title/Abstract] OR Diabetes[Title/Abstract] OR Dyslipidemia[Title/Abstract] OR *Cholesterol*[Title/Abstract] OR *Triglyceride*[Title/Abstract] OR Obesity[Title/Abstract] OR *Glycemia*[Title/Abstract] OR *Statin*[Title/Abstract]) AND (Tendinitis[Title/Abstract] OR "Tendon Disorder*"[Title/Abstract] OR "Tendon Inflammation"[Title/Abstract] OR Tendinopath*[Title/Abstract]) Filters: from 1000/1/1 - 2024/3/31

Scopus database: (1081 results)

( TITLE-ABS-KEY ( ( "metabolic diseases" ) OR ( "metabolic alterations" ) OR ( diabetes ) OR ( dyslipidemia ) OR ( *cholesterol* ) OR ( *triglyceride* ) OR ( obesity ) OR ( *glycemia* ) OR ( *statin* ) ) AND TITLE-ABS-KEY ( "tendinitis" ) OR TITLE-ABS-KEY ( tendinopath* ) ) AND PUBYEAR < 2024 OR PUBDATETXT( "January 2024" OR "February 2024" OR "March 2024")

Web of science: (394 results)

TS=( ( (“Metabolic Diseases”) OR (“Metabolic Alterations”) OR (Diabetes) OR (Dyslipidemia) OR (*Cholesterol*) OR (*Triglyceride*) OR (Obesity) OR (*Glycemia*) OR (*Statin*)) AND ((Tendinitis) OR ( “Tendon Disorder*”) OR (“Tendon Inflammation”) OR (Tendinopath*)))

refined with Filter for Publication Date: from 1950-01-01 up to 2024-03-31

Supplementary tables for raw data extraction of included studies are provided here:

https://osf.io/bsxta/?view_only=948d3bbb5ead4ba897dd3a3801039a51
